# The Onset of Molecule‐Spanning Dynamics in Heat Shock Protein Hsp90

**DOI:** 10.1002/advs.202304262

**Published:** 2023-11-20

**Authors:** Benedikt Sohmen, Christian Beck, Veronika Frank, Tilo Seydel, Ingo Hoffmann, Bianca Hermann, Mark Nüesch, Marco Grimaldo, Frank Schreiber, Steffen Wolf, Felix Roosen‐Runge, Thorsten Hugel

**Affiliations:** ^1^ Institute of Physical Chemistry University of Freiburg Albertstrasse 21 79104 Freiburg Germany; ^2^ Institute of Applied Physics University of Tübingen Auf der Morgenstelle 10 72076 Tübingen Germany; ^3^ Science Division Institut Max von Laue ‐ Paul Langevin 71 avenue des Martyrs Grenoble 38042 France; ^4^ Department of Biochemistry University of Zurich Winterthurerstrasse 190 CH‐8057 Zurich Switzerland; ^5^ Biomolecular Dynamics, Institute of Physics University of Freiburg Hermann‐Herder‐Strasse 3 79104 Freiburg Germany; ^6^ Department of Biomedical Sciences and Biofilms‐Research Center for Biointerfaces (BRCB) Malmö University 20506 Malmö Sweden; ^7^ Division of Physical Chemistry Lund University Naturvetarvägen 14 22100 Lund Sweden; ^8^ Signalling Research Centers BIOSS and CIBSS University of Freiburg Schänzlestrasse 18 79104 Freiburg Germany

**Keywords:** heat shock protein 90, molecular dynamics simulations, neutron scattering, protein dynamics, single molecule fluorescence

## Abstract

Protein dynamics have been investigated on a wide range of time scales. Nano‐ and picosecond dynamics have been assigned to local fluctuations, while slower dynamics have been attributed to larger conformational changes. However, it is largely unknown how fast (local) fluctuations can lead to slow global (allosteric) changes. Here, fast molecule‐spanning dynamics on the 100 to 200 ns time scale in the heat shock protein 90 (Hsp90) are shown. Global real‐space movements are assigned to dynamic modes on this time scale, which is possible by a combination of single‐molecule fluorescence, quasi‐elastic neutron scattering and all‐atom molecular dynamics (MD) simulations. The time scale of these dynamic modes depends on the conformational state of the Hsp90 dimer. In addition, the dynamic modes are affected to various degrees by Sba1, a co‐chaperone of Hsp90, depending on the location within Hsp90, which is in very good agreement with MD simulations. Altogether, this data is best described by fast molecule‐spanning dynamics, which precede larger conformational changes in Hsp90 and might be the molecular basis for allostery. This integrative approach provides comprehensive insights into molecule‐spanning dynamics on the nanosecond time scale for a multi‐domain protein.

## Graphical abstract

Protein dynamics occur on a wide range of timescales and are key for protein functionality. An unprecedented combination of single‐molecule fluorescence, quasi‐elastic neutron scattering and all‐atom molecular dynamics simulations reveals surprisingly fast molecule‐spanning dynamics around 150 ns in the heat shock protein 90 (Hsp90). They depend on the conformational state of Hsp90 and are likely precursors for larger (allosteric) conformational changes.

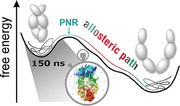

## 1 Main

The complexity of understanding protein function results from the involvement of dynamic processes occurring on a broad range of time scales.^[^ ^1^ ^]^ These processes are connected to different length scales ranging from local structural dynamics and substrate processing, for example, adenosine triphosphate (ATP) hydrolysis, to long‐range coupled structural or dynamical changes, that is, allostery.^[^ ^2^, ^3^, ^4^, ^5^, ^6^ ^]^ Numerous studies have provided important insights into dynamics on the µs to sec time scale. Protein binding kinetics and transitions between defined conformational states have been successfully investigated.^[^ ^7^, ^8^, ^9^, ^10^, ^11^, ^12^ ^]^ However, it has become more and more clear that the dynamics within conformational states, likely on time scales of a few to several hundreds of nanoseconds, are also crucial, as these might eventually enable and drive conformational transitions.^[^ ^13^, ^14^, ^15^, ^16^ ^]^

**Figure 1:**
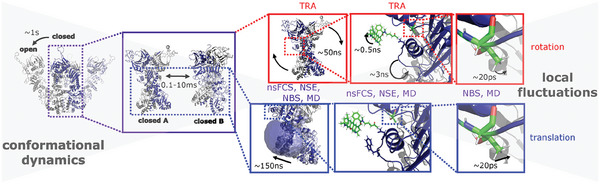
Comprehensive picture of Hsp90 dynamics. Here, we complete this picture by accessing the 150 ns time scale, which we show to unravel molecule‐spanning dynamics. Disentanglement of rotational and translational dynamics (red and blue frames, respectively) is feasible by combining nanosecond fluorescence correlation spectroscopy (nsFCS), time‐resolved anisotropy (TRA), neutron spin echo spectroscopy (NSE), neutron backscattering spectroscopy (NBS) and molecular dynamics (MD) simulations. The time scales (from experiments) of the respective dynamics are given in the boxes. TRA is only sensitive to rotations, while all other methods are sensitive to rotation and translation.

Here, we address this fundamental challenge using the well‐established multi‐domain protein Hsp90 (see **Figure** **1**). Hsp90 is a central regulatory element in various cellular processes such as the maturation of kinases or steroid hormone receptors, to name only a few.^[^ ^17^, ^18^, ^19^ ^]^ In its function as a molecular chaperone, Hsp90 helps many of its several hundred clients to develop their native, biologically active conformation. Other clients, such as the aforementioned kinases, rely on the continuous binding to Hsp90 to maintain an active conformation making Hsp90 a mediator for signal transduction.^[^ ^17^, ^18^, ^19^, ^20^ ^]^ Misfolded proteins and derogation of signaling pathways are often linked to severe neurodegenerative diseases and cancer, which makes Hsp90 a promising drug target.^[^ ^21^ ^]^ Throughout its chaperoning progression, several co‐chaperones assist Hsp90 in client maturation by modulation of its ATPase activity. Sba1, the yeast homolog of human p23, is such a functionally important co‐chaperone that is directly involved in nucleotide‐dependent stalling of Hsp90 conformational states, thereby supporting client control.^[^ ^22^, ^23^, ^24^, ^25^ ^]^

Yeast Hsp90 (Hsp82) is a homodimer with each monomer having a molecular weight of 82 kDa and consisting of 709 amino acids arranged in three domains: The C‐domain, responsible for dimerization, the M‐domain, hosting important co‐chaperone and client binding sites and the N‐domain, harboring a nucleotide binding site as well as co‐chaperone and client binding sites.^[^ ^26^ ^]^ Throughout its chaperoning cycle, Hsp90 transitions between open and closed conformations on time scales from milliseconds to several minutes that are well investigated.^[^ ^19^, ^27^, ^28^, ^29^, ^30^, ^31^ ^]^ Also local dynamics on the picosecond time scale have been reported before for Hsp90.^[^ ^32^, ^33^ ^]^ To complete the picture of a continuous dynamical progression throughout the Hsp90 cycle the nanosecond time scales still need to be addressed. Linking spatial information on short time scales to global conformational changes will help to decipher the mechanistic origin of the function‐dependent molecular flexibility of this system and to better understand the molecular basis of the transitions between conformations.

Given the complexity of attempting to reliably characterize nanosecond protein dynamics of Hsp90, we use an integrative approach combining several experimental techniques and computer simulations (outlined in Figure S1, Supporting Information). Fluorescence correlation spectroscopy (FCS) probes correlated dynamics based on characteristic decays or rises of the fluorescence intensity correlation function.^[^ ^34^, ^35^ ^]^ In the last decade, the potential of FCS on the nanosecond time scale was successfully demonstrated by studying unfolded proteins.^[^ ^36^, ^37^, ^38^, ^39^, ^40^ ^]^ Important pre‐requisites for clear data interpretation are the separation of time scales^[^ ^41^ ^]^ and the complementary information from single‐molecule Förster resonance energy transfer (FRET). Labeling with FRET donors and acceptors provides additional information by cross‐correlating the donor and acceptor signals.^[^ ^42^ ^]^ Significant anti‐correlations were observed for small intrinsically disordered proteins (IDPs), but representative nsFCS studies of large structured proteins are scarce.^[^ ^39^, ^43^, ^44^ ^]^ Despite the significant importance of multi‐domain proteins as regulators, the usefulness of nsFCS for these proteins so far is still elusive.

Time‐resolved anisotropy (TRA) probes rotational dynamics of dye‐coupled biomolecules by time‐dependent and polarization‐sensitive detection of fluorescence intensities.^[^ ^45^ ^]^ Time‐correlated single photon counting (TCSPC) enables studying the rotation of labeled proteins as for example shown for small IDPs.^[^ ^11^, ^46^ ^]^ In multi‐domain proteins, the situation is slightly more complex because multiple rotational modes exist. It is not clear a priori, how the signals from independent effects are superimposed and can be disentangled. If the timescales are clearly separated (more than a factor of 10), a multi‐exponential fit can be used.^[^ ^43^ ^]^ In other cases more sophisticated fit models might be necessary, as for example the *cone‐in‐cone* model.^[^ ^47^ ^]^ With pulsed interleaved excitation (PIE) stoichiometries can be determined, which further supports species‐specific analysis. Together with polarization‐resolved nsFCS data, rotational and conformational contributions can be disentangled.^[^ ^43^ ^]^

Quasi‐elastic neutron scattering (QENS) simultaneously accesses spatial and time correlations by probing the scattering function depending on the momentum ℏ*q* and energy transfer ℏω of the neutron during the interaction with the sample. QENS constitutes a label‐free, non‐invasive, and non‐destructive technique that measures an ensemble‐averaged signal with an unambiguous interpretation in terms of statistical mechanics. Most importantly, the QENS signal contains information on the diffusive motions, which, due to their dependence on *q*, can be directly associated with length scales of structural motions and spatial confinement. Based on the distinction between so‐called coherent and incoherent scattering associated with the neutron and nuclear spin statistics involved in the scattering, both self‐ (tracer) and collective (mutual) diffusion processes can be quantified. Early work on proteins focused on dynamics in hydrated powders to target aspects of the water‐protein coupling.^[^ ^48^, ^49^ ^]^ Recent advances in neutron instrumentation allow us to obtain signals from protein solutions that are sufficiently strong to separate the different superimposed contributions from localized as well as rotational and translational center‐of‐mass diffusive motions of proteins in aqueous solutions.^[^ ^50^ ^]^ Importantly, with neutron spin echo (NSE) we access these molecule‐spanning multi‐domain motions unambiguously on well‐defined nanosecond time and nanometer length scales.^[^ ^50^, ^51^, ^52^, ^53^, ^54^ ^]^

We complement our data interpretation with full‐atom molecular dynamics (MD) simulations, which are ideal to probe molecular details on these fast time scales, as time scales of hundreds of nanoseconds are readily accessible even for large proteins with sufficient sampling. We use MD simulations to investigate both local details such as the free volume at specific sites as well as molecule‐spanning dynamic modes.^[^ ^55^ ^]^ The measured dynamics are neither purely Brownian dynamics nor can they be described by a simple harmonic oscillator. We access a complex pattern of diffusive modes, which can be described with our integrative approach covering a wide range of time and length scales. Overall, together with the previously published dynamics on slower time scales^[^ ^31^, ^56^, ^57^ ^]^ our results complete a comprehensive picture of Hsp90's dynamics from the nanosecond to second time scale.

Several investigations have shown that fast dynamics on the picosecond to nanosecond time scale are functionally important for many biochemical processes.^[^ ^43^, ^58^, ^59^ ^]^ The importance of dynamics on slow time scales of milliseconds and longer are obvious, because they define the occupancy of functional states, as for example, the N‐terminal open and N‐terminal closed state of Hsp90.^[^ ^19^ ^]^ Less clear is a possible functional relevance of dynamics on the 150 ns time scale. As a first step toward understanding the relevance of these dynamics, we investigated if its time scale depends on the functional state of Hsp90. In addition, we scrutinize if the binding of Sba1, a functionally important co‐chaperone of Hsp90 (see above), affects the molecule‐spanning nanosecond dynamics of Hsp90. Interestingly, the open state molecule‐spanning nanosecond dynamics significantly differ from the closed state one, while Sba1 binding affects the dynamics in a location‐dependent manner.

This article is structured as follows: i) nsFCS experiments show a bunching term around 150 ns. ii) A combination of nsFCS, fluorescence anisotropy decay experiments, MD simulations and rigid body calculations shows that this term is not caused by global rotations, which occur around 60–80 ns. iii) We interpret the bunching term at around 150 ns as global conformational changes of the protein, which is supported by MD simulations. These conformational changes lead to changes in the accessible volume (AVs) of one or both dyes (as also confirmed by MD simulations) and therefore fluctuations in the fluorescence signals. iv) MD simulations show that these correlations are caused by several molecule‐spanning modes within Hsp90. v) Such internal global dynamics are also seen in NSE experiments. vi) Finally, the state specificity and the effect of the co‐chaperone Sba1 on these molecule‐spanning dynamics is investigated.

## 2 Results and Discussion

### 2.1 nsFCS Reveals Hsp90 Dynamics on the 150 ns Time Scale

**Figure 2:**
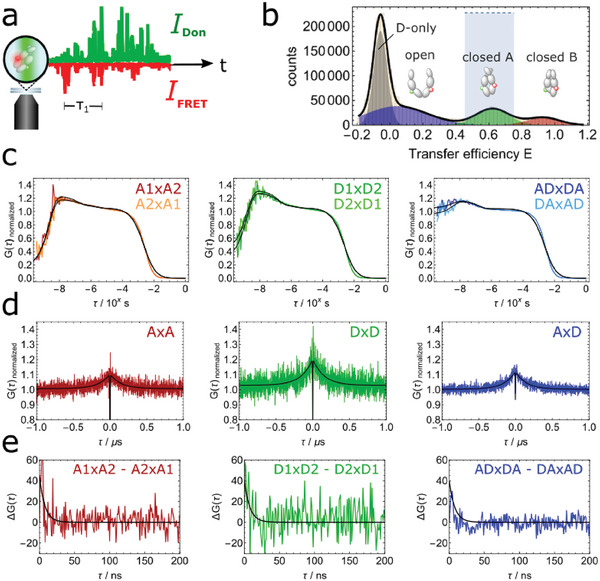
Substate‐specific nsFCS analysis of the Hsp90 FRET pair 452‐452 labeled with Atto550 and Atto647N with AMPPNP. The results of all six independent measurements are given in Table S1, Supporting Information. Exemplary data from one measurement is shown here. a) Scheme of a nsFCS experiment. b) Substate‐selection based on the FRET efficiency *E*. Selecting single‐molecule events with *E* = 0.4–0.8 (light blue part) enables specific analysis of the closed A conformation of Hsp90. c) Logarithmic substate‐specific polarized‐FCS curves from seconds to picoseconds correlating the parallel and the perpendicular detection signal and vice versa. Fit models include diffusion, bunching and antibunching components, for the autocorrelations (D×D and A×A) additionally a triplet kinetics component was used. d) Linear substate‐specific nsFCS curves. From a global analysis of all three channel correlations (D×D, A×D, and A×A) we obtain a correlation time of (149  ±  7) ns. For the complete nsFCS analysis see Table S1, Supporting Information. Errors were determined as standard errors of the mean for the six independent measurements. e) Subtraction of the two not normalized nsFCS branches shows the influence of polarization and therefore rotation on the observed dynamics. Shown in black are double exponential decays with relaxation times of 3 and 60 ns. Both are much shorter than the 149 ns dynamics observed in (d).

nsFCS experiments were performed on a homebuilt setup (see Experimental Section and Figure S2, Supporting Information) with two FRET pairs, one spanning the M‐domain of the Hsp90 dimer (452‐452) and the other one the N‐domain of the Hsp90 dimer (61‐61). For a graphical illustration of the labeling positions we refer the reader to Figure 4b. While FRET pairs with position 452 report on dynamics across the lumen of the Hsp90 dimer, which is relevant for substrate and co‐chaperone binding,^[^ ^19^ ^]^ we chose position 61 because it reports on the dynamics of the N‐terminal domains.^[^ ^29^ ^]^ Positions 61 and 452 can be labeled with fluorophores and are still functional as has been shown before.^[^ ^60^ ^]^ All measurements in **Figure** **2** were conducted in the presence of 2 mm AMPPNP, a non‐hydrolizable ATP analog, to populate the N‐terminal closed conformational states of Hsp90. Figure 2c shows a representative FCS data set for the FRET pair 452‐452 with AMPPNP spanning the nanosecond to second time scale. For the data on the FRET pair 61‐61 see Figure S3, Supporting Information. We selected the closed state A (AMPPNP‐bound state^[^ ^56^ ^]^) of Hsp90 from the FRET efficiency histogram for a substate specific nsFCS analysis (Figure 2b), the open state is discussed below. The auto‐ and crosscorrelations shown in Figure 2d show a photon antibunching component with a decay time of ≈3 ns which can be attributed to the fluorescence lifetime of the dyes (see Figure S4, Supporting Information, for lifetime data). In addition, we see a clear bunching component at around 150 ns. The ≈ 150 ns component is present in both the autocorrelations (Don×Don and FRET×FRET) as well as in the cross‐correlation (Don×FRET). Fitting the auto‐ and crosscorrelations globally (all three curves have the same decay time) of six independent nsFCS experiments results in the average bunching time of (149 ± 7) ns, the error represents the standard deviation of the six independent measurements. Because the cross‐correlation does not show a clear anticorrelated behavior at the time scale of 150 ns, a directly opposed structural movement between the investigated positions cannot be the reason for this signal.^[^ ^42^ ^]^ More likely, the correlation is caused by internal dynamics of Hsp90 subdomains, rotation of the overall protein or a superposition thereof. Such dynamics could for example affect the brightness of the donor and therefore show a correlation in all three signals. This is further investigated below with the help of MD simulations.

**Figure 3:**
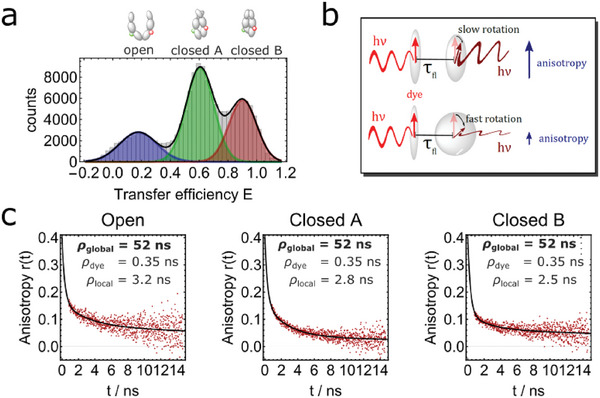
Substate‐specific time‐resolved single‐molecule anisotropy of the Hsp90 FRET pair 452‐452 with AMPPNP. a) The transfer efficiency histogram reveals three conformational states of Hsp90: the open state (*E* ≈ 0.17, highlighted blue), closed state A (*E* ≈ 0.6, highlighted green), and closed state B (*E* ≈ 0.9, highlighted red). For the following graphs, donor‐only and acceptor‐only molecules were excluded by selecting exclusively single‐molecule events via dual channel burst search algorithm for further analysis. b) Schematic view of an anisotropy experiment. Fluorescence depolarization depends on the lifetime and rotation of the excited dye dipole and is measured by polarization‐sensitive detection. c) Acceptor anisotropy decay upon direct acceptor excitation at Hsp90 FRET pair 452‐452 for the three different populations. In black a *cone‐in‐cone* model fit is shown. *r*_0_ = 0.4 and global parameters ρ_dye_ and ρ_global_ result in a rotational decay time of (52  ±  32) ns. Fit results and standard errors for all sub‐populations are shown in Table S2, Supporting Information. Figure S5 and Table S6, Supporting Information, show the results for other fit functions that give comparable values.

**Figure 4:**
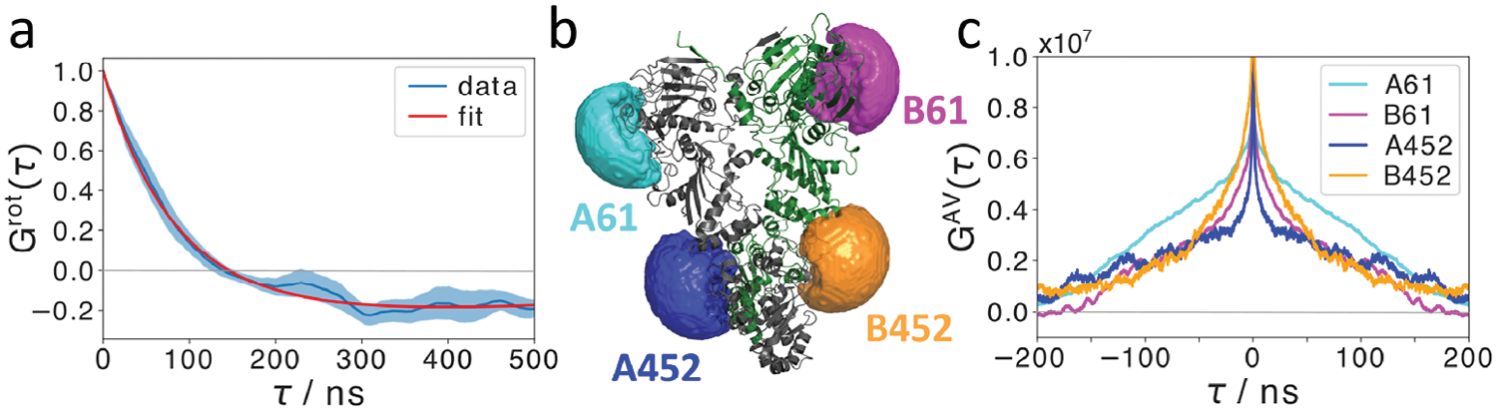
MD simulations confirm rotational correlation and provide a link between nsFCS and dye accessible volumes. a) Autocorrelation of Hsp90's first principal axis of the moment of inertia orientation reveals a rotational correlation time of (81 ± 1) ns. Shaded area depicts the standard error of the mean for five statistically independent simulations. b) A representative MD snapshot of the Hsp90 dimer visualizes the investigated accessible dye volumes (colored spheres). Hsp90 chains A and B are colored gray and green, respectively. c) Autocorrelation functions of accessible dye volumes at Hsp90 positions 61 and 452 at chain A or B, respectively. Unconstrained bi‐exponential fits reveal correlation times on the 100 ns time scale, which might be the cause for fluctuations in fluorescence intensity resulting from structural dynamics (see Figure S6, Supporting Information).

### 2.2 Separating Rotation from Internal Dynamics by nsFCS and Time‐Resolved Anisotropy

To clarify the origin of this 150 ns dynamics, we plot the two branches of the autocorrelation functions on top of each other (Figure 2c). One branch shows the correlation of the horizontal with the vertical polarized signal and the other branch the vertical with the horizontal polarization. If there were rotational contributions, these should show up in a difference between the two (normalized) branches.^[^ ^61^ ^]^ Indeed, we observe some deviation on time scales below 100 ns, which hints toward rotation on the sub‐100 ns time scale. To quantify this difference, we plot and fit the difference between the two correlations in Figure 2e). The exponential decays show relaxations times ≈3 ns, which is consistent with the fluorescence lifetime of the dye, and a slower component of around 60 ns, which is attributed to the global rotation of the Hsp90 dimer and will be further investigated with anisotropy decay experiments, MD simulations and rigid body approximations below.

**Figure** **3**a shows single‐molecule fluorescence data of the FRET pair 452‐452 with AMPPNP. Three conformational states can be distinguished by FRET efficiency *E* versus stoichiometry *S* analysis: An open state at *E* ≈ 0.13, a closed state at *E* ≈ 0.63 and a more contracted closed state at *E* ≈ 0.93. This is consistent with previous results^[^ ^56^ ^]^ and with the independent nsFCS experiments (Figure 2). FRET efficiency and stoichiometry were used as selectors to perform subpopulation‐specific anisotropy analysis. Figure 3c shows the anisotropy decays for the open, closed state A and closed state B at position 452. To describe the data we used the *cone‐in‐cone* model which accounts for free dye rotation ρ_dye_, dynamics of the local environment ρ_local_ and for global rotation of the overall protein ρ_global_ (see Experimental Section, Equation (9)).^[^ ^47^ ^]^ A summary of all fit results is shown in Table S2, Supporting Information. We obtain ρ_dye_ at (0.35 ± 0.05 ns) which is comparable to the rotational correlation coefficient obtained for freely diffusing dyes,^[^ ^62^ ^]^ local rotational time ρ_local_ population depended at ≈ 3 ns and ρ_global_ at (52  ±  32) ns. The rotational times are faster than the bunching term of around 150 ns of the nsFCS data, supporting the finding from the nsFCS experiments, namely that rotation does not significantly contribute to the dynamics on the 150 ns time scale. Altogether, our experiments disfavor global rotation as the main cause of the correlation on the time scale of ≈ 150 ns and prompts us to propose a significant contribution from global internal Hsp90 dynamics.

### 2.3 MD Simulations Show Internal Hsp90 Dynamics

#### 2.3.1 MD Simulations Result in Rotational Correlation Times Similar to Anisotropy Decays

To further investigate the rotation of the Hsp90 dimer, we characterized the rotational diffusion from atomistic MD simulations by calculating the autocorrelation function of the first principal axis of inertia. The movement of the first principal axis of the moment of inertia, that is, the longest axis through the protein if it were approximated by an ellipsoid and which is similar to the axis along the dimer interface, is obtained from 5 × 1 µs MD simulations of the full Hsp90 dimer with AMPPNP as depicted in **Figure** **4**a. A single exponential fit results in a decay time of 47 ± 1 ns, but reproduced the autocorrelation function poorly (*R* ^2^ = 0.76). We therefore use the equation

(1) $$G^{\mathrm{rot}}\left(\tau \right)=A\exp\left(-\tau /\tau_{1}\right)+B\cos\left(2\pi \omega \tau +\theta \left(\phi \right)\right)$$

with a rotation frequency ω and phase angle θ(ϕ) to account for contributions from potentially continuous rotation owing to simulation starting conditions that still persist after 2 µs simulation time. The respective fit exhibits good agreement with *G* ^rot^(τ) (*R* ^2^ = 0.99) and results in a decay time constant τ_1_ = 81 ± 1 ns, which ends up in good agreement with both the nsFCS and TRA experiment. Concerning the amount of persistent rotation, we find that the weight factors are *A* = 1.17  ±  0.02 and *B* = 0.199  ±  0.002. The ratio of rotational diffusion versus continuous rotation is therefore about 6:1.

#### 2.3.2 MD‐based Accessible Dye Volume Correlations Reveal Structural Hsp90 Dynamics

To come to a possible explanation for the observed correlation of around 150 ns in the nsFCS data, in the following we calculate the fluctuations in the accessible volume (AV) of the dyes from MD simulations. Changes in AVs over time result in changes in the fluorescence of dyes^[^ ^63^, ^64^ ^]^ and therefore a signal in nsFCS, which is further discussed in the following. Complementary to TRA experiments, which selectively probe rotational dynamics, MD simulations can provide access to local dynamics of Hsp90 to disentangle them from global rotational diffusion. Therefore, we calculated the AV as a scalar value for each simulation snapshot (i.e., with a 100 ps time resolution) at Hsp90 positions which were investigated by nsFCS, namely amino acid numbers 61 and 452 (see Figure 4b for an illustration), and then computed their ensemble‐averaged, time‐dependent autocorrelation functions (Figure 4c) to investigate position‐specific AV changes on characteristic time scales.

We obtain correlation curves exhibiting two dominant decays. Unconstrained bi‐exponential fits reveal a fast correlation below 100 ps, which is faster than the minimum lag time and therefore are not further discussed here. More interestingly, all positions show a slower correlation on the 100 ns time scale (see Figure 4b; Figure S6 and Table S3, Supporting Information for a summary of all fits). A likely scenario that explains our observed nsFCS time scales is that structural fluctuations confine the dye flexibility. This confinement might be caused by side chain dynamics or the movement of domains. From the fluorescence viewpoint, such a confinement changes the properties of the dye. A change in the accessible volume can change the mean position of the dye or the probability of collision with neighboring side chains and thus result in a higher possibility of quenching. Altogether our data indicates that the latter mechanism is prevalent here (see Section S5 and Figure S16, Supporting Information), in particular, because we see a reduction of the lifetime of the donor in the nsFCS data (Table S1, Supporting Information) and in lifetime control experiments (Table S5, Supporting Information). These observations provide a connection between nsFCS and simulations and support our hypotheses that structural dynamics contribute to the observed ≈ 150 ns FCS time scale. Interestingly, the simulation‐based AV cross‐correlations show an anticorrelated component, as well, with amplitudes that are about one order of magnitude weaker than those of the AV autocorrelations and about the same time scale of 100 ns (Figure S6, Supporting Information). In the experiment, a superposition of these signals is detected, which likely explains why we do not see the anti‐correlated signal there. We cannot exclude additional contributions to the nsFCS signals, but the good agreement for several dye positions supports that changes in the AV are the main contribution in our case. In the future, further correlation and lifetime analysis similar to Schulze et al.^[^ ^65^ ^]^ could shine more light on the detailed quenching mechanism, but this will be more complicated here, because likely not only a single quenching amino acid is involved (see Figure S16, Supporting Information). We conclude that combining nsFCS with information from TRA and MD simulations is a versatile strategy to disentangle rotational from internal dynamics, which puts us in the ideal situation to compare them to results from complementary techniques such as neutron spin echo (see below).

#### 2.3.3 Cartesian PCA Reveals Molecule‐spanning Dynamics of the full Hsp90 dimer

To gain more information on the Hsp90 dimer‐internal timescales and their locations, we now address a cartesian principal component analysis (PCA) of all backbone and C_β_ atoms from one representative of the five 1 µs simulations (Figure S7, Supporting Information). The eight eigenvectors that cover 80% of the overall positional variance all represent molecule‐spanning motions of the full dimer, as can be seen from the root mean square fluctuation (RMSF) per atom and eigenvector along the protein chain. The time traces of projections per eigenvector and their autocorrelation functions (ACFs) show that no clear separation between slow or fast modes exists. Instead, a continuum of time scales between tens and several hundreds of nanoseconds appears. This continuum does not stand in contrast to the well‐defined time scale observed in nsFCS. The fluorescence correlation time depends on the time scale of changes in dye‐accessible volumes, which are directly caused by local structural changes. Our integrative approach shows that these local changes are caused by global structural dynamics. The first two eigenvectors (1 and 2) contain the majority of variance in motions (≈ 60 % of total variance), that is, they are the largest motions. The ACF analysis reveals that they constitute the slowest motions, as well. The respective structures of these projections along eigenvectors 1 and 2 over time are shown in Movies [Link], [Link], [Link], [Link], Supporting Information. While morphing between the states with maximal projection values (Movies S1 and S2, Supporting Information) highlights the delocalized nature of these motions. We want to emphasize that the eigenvectors calculated here do not encode low frequency harmonic vibrations, but a highly diffusive and fluctuating bending‐contraction motion, as can be seen in the actual time series of projections (Movies S3 and S4, Supporting Information).

#### 2.3.4 Diffusion from Rigid Body Calculations

In order to validate the simulation results in terms of absolute time scales, we estimated the translational and rotational diffusion coefficient for the average protein structures of the five independent MD runs. To this end, we employed hydrodynamic bead modeling via the software package HYDROPRO^[^ ^66^ ^]^ resulting in the diffusion tensor of a rigid body, that is, neglecting any internal dynamics. The resulting translational diffusion coefficient of $D_{t}^{\text{(rigid)}}=\left(4.71\pm 0.04\right)\times \;10^{-7}$ cm^2^ s^−1^ (20 °C in water, mean ± SD from the five simulation structures) is in good agreement with the translational diffusion coefficient $D_{t}^{\text{(MD)}}=\left(4.4\pm 0.5\right)\times \;10^{-7}$ cm^2^ s^−1^ from the MD simulations. The rotational diffusion coefficient yields $D_{r}^{\text{(rigid)}}=\left(1.64\pm 0.03\right)\times \;10^{6}$ s^−1^ (20 °C in water), which corresponds to a mean rotational time of τ_r_ = 1/(6*D*_r_) = (101 ± 2) ns. However, the anisotropy of the molecule results in rotational times of (72 ± 2) ns around the first principal axis, and (125 ± 5) ns around the other two principal axes. We remark that this anisotropic variation of rotation times might explain the slight difference between observed times in MD simulations and TRA, as different techniques probe different combinations of these time scales.

### 2.4 NSE Shows a Global Internal Diffusive Mode on Nanosecond Time Scales

**Figure 5:**
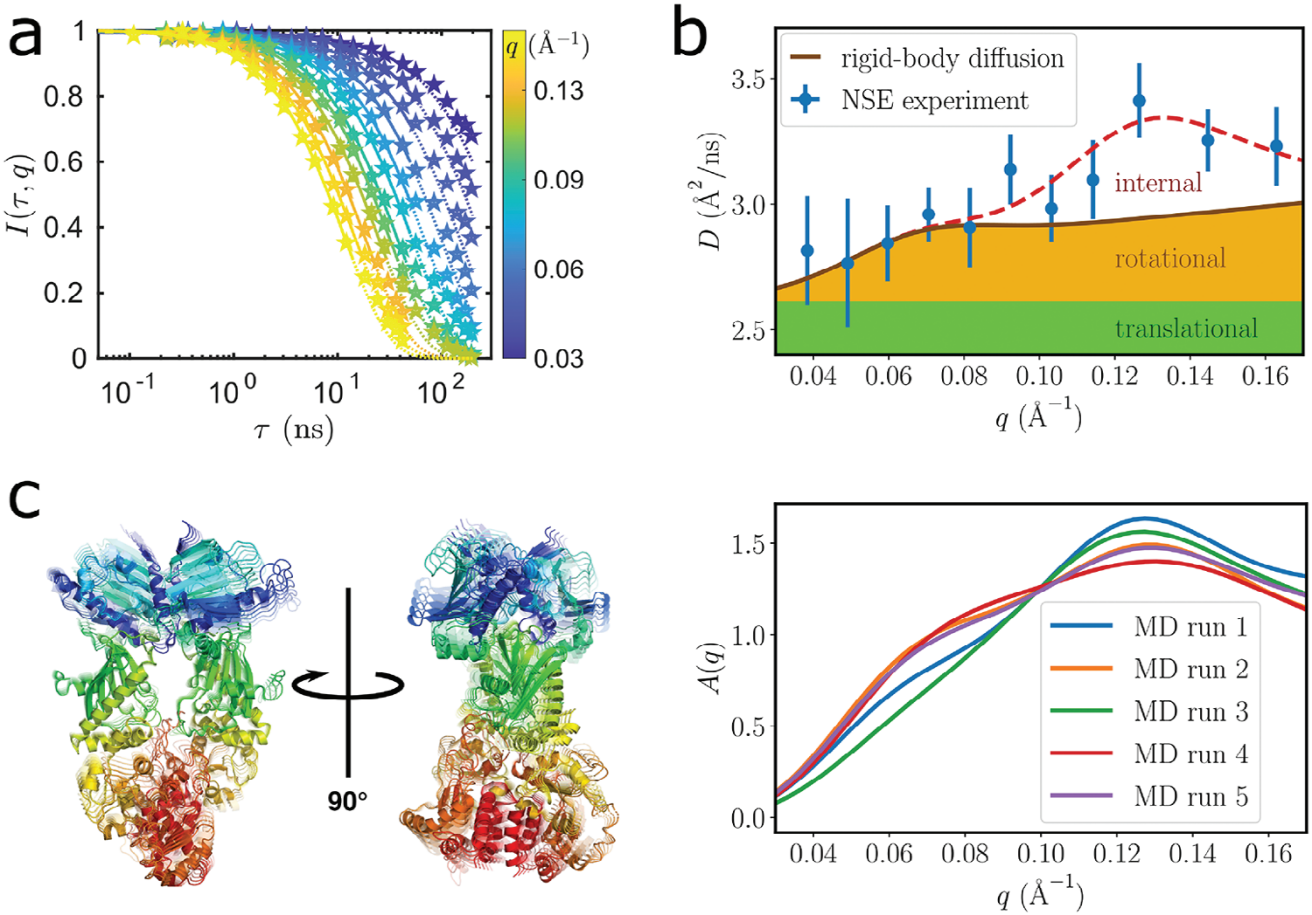
Neutron spin echo spectroscopy shows internal motions beyond the translation and rotation of the entire molecule. a) Fits of the intermediate scattering functions for different scattering vectors *q* (color coded). The fits (solid lines) were performed using Equation (2) for τ < 30 ns and for *I*(*q*, τ) > 0.3. They were extrapolated with dotted lines to outline the expected deviation from the single exponential suggesting the presence of internal motions. b) The resulting experimental diffusion function *D*(*q*) evidences a first shoulder around the scattering vector *q* ≈ 0.07 Å^−1^ and a peak around *q* ≈ 0.13 Å^−1^. While the shoulder can be described by rotational diffusion of the entire protein based on a rigid‐body modeling, the peak originates from an internal degree of freedom. c) Visualization of the first and most relevant principal component of Hsp90: a bending motion with a slight twist spans over the entire molecule (left). Calculated amplitude functions *A*(*q*) based on Equation (3) signatures of the ten PCA eigenvectors obtained from five MD runs with independent start parameters (see main text for details). The peak around *q* ≈ 0.13 Å^−1^ indicates that Hsp90 performs concerted internal motions on times scales of 100 ns (right), consistent with nsFCS.

We now assess if such global internal diffusive modes on the hundred nanosecond time scale can be seen in neutron spin echo (NSE) spectroscopy. NSE experiments provide unique information on the collective motions inside proteins^[^ ^50^, ^51^, ^52^ ^]^ and are therefore complementary to the fluorescence experiments. The initial decay of the experimental intermediate scattering function *I*(*q*, τ) (**Figure** **5**a) was determined by a single exponential fit to the initial slope (τ < 30 ns and *I*(*q*, τ) > 0.3) for each value of the scattering vector *q* individually

(2) $$I\left(q,\tau \right)=C\exp\left(-D_{\mathrm{eff}}q^{2}\tau \right)$$

with a scalar prefactor *C* ≈ 1. The resulting effective short‐time diffusion function *D*_eff_(*q*) (Figure 5b) encodes the relaxation of density correlations, and is thus related to the inhomogeneity of the protein structure in solution: Correlations on large length scales beyond the size of the protein can only relax via translational diffusion, while correlations on shorter length scales within the protein relax faster due to a combination of translational and rotational diffusion and internal diffusive motions such as multi‐domain dynamics and bending.^[^ ^50^, ^67^ ^]^

Two main features are observed. At lower *q* ≈ 0.07 Å^−1^ corresponding to length scales of ≈ 10 nm, *D*_eff_(*q*) shows a shoulder. At higher *q* ≈ 0.13 Å^−1^ corresponding to length scales of ≈ 4.8 nm, a peak is observed.

In order to model the different contributions, we started with the effects arising from rigid‐body translational and rotational diffusion (solid line in Figure 5b, for details, see Section S3.4, Supporting Information). The absolute values of *D*_eff_ start at the translational diffusion coefficient in the low *q* limit, and then increase due to contributions of rotational diffusion on *q* values corresponding to the entire protein size. The absolute value of *D*_eff_ does not quantitatively represent a pure dimer solution (see Figure S8, Supporting Information), therefore we used a hypothetical hexamer solution by rescaling the translational and rotational diffusion coefficient based on their relation to the protein radius as $D_{t}^{\left(\text{hex}\right)}=D_{t}^{\left(\text{dimer}\right)}/\sqrt[{3}]{3}$ and $D_{r}^{\left(\text{hex}\right)}=D_{r}^{\left(\text{dimer}\right)}/3$. NSE experiments require very high concentrations of proteins, even higher than NMR experiments. Therefore, the state of Hsp90 in our experiments is self‐consistent: At very low concentrations (fluorescence experiments) it is dimers, at higher concentrations (and in Figure S8, Supporting Information), dimers and hexamers can be seen. Clusters are likely transient, such as that they do not fully show in SANS.^[^ ^50^ ^]^ Nevertheless, in SANS, there is an indication of the presence of some larger objects (Figure S9, Supporting Information) The transient hexamer state is thus likely caused by crowding, as is the case for many other proteins^[^ ^68^, ^69^, ^70^ ^]^ and does not contradict the SEC result (Figure S10, Supporting Information). Cluster formation and crowding have been shown to have little effect on the internal dynamics on the neutron spectroscopy observation scale in earlier work,^[^ ^50^, ^71^ ^]^ but may nevertheless be different. Finally, Lee et al. have shown that the building block of the hexameric assembly is a dimer, again supporting that we measure the internal dynamics of a dimer.^[^ ^72^ ^]^

The approximative modeling based on an oligomeric state recovers the shoulder at *q* ≈ 0.07 Å^−1^ well, but clearly fails to explain the peak feature at *q* ≈ 0.13 Å^−1^, and in general higher values of *D*_eff_(*q*) at larger *q*. This deviation is shown as a dashed guide to the eye in Figure 5b and is indicative of additional internal motions of the protein, as they occur at *q* values corresponding to motions within the protein. We stress that the significance of this additional contribution is independent of the assumption of oligomers.

For more detailed modeling of the internal motions, we based calculations on the cartesian principal component (PC) analysis from the MD simulations. As the central observable, an amplitude function *A*_*k*_(*q*) is obtained for the *k* ^th^ eigenvector:^[^ ^50^, ^51^ ^]^

(3) $$A_{k}\left(q\right)=\sum_{\alpha ,\beta }b_{\alpha }b_{\beta }\exp\left(iq\cdot (r_{\alpha }-r_{\beta })\right)\left(\hat{q}\cdot {\hat{v}}_{k\alpha }\right)\left(\hat{q}\cdot {\hat{v}}_{k\beta }\right)$$

where the sum runs over all atom pairs with index α and β with coordinates **r_x_**. *b*_*x*_ denote scattering cross‐sections of the atoms. The cartesian PC eigenvector provides displacements vectors ${\hat{v}}_{\mathrm{kx}}$ at each atom position, which together with the scattering vector **q** allow for the geometric interpretation of collective structural dynamics of multi‐domain proteins. The first ten PC eigenvectors were used for further analysis. We weighted the amplitudes according to the square‐root of the corresponding PC eigenvalues, and finally obtained the expected experimental signature of the internal motions present in MD simulations (Figure 5c). The calculated *A*(*q*) and the experimental *D*(*q*) exhibit good agreement in their line shape. Importantly, all five independent runs of MD simulations show a consistent peak at *q* ≈ 0.13 Å^−1^, which unambiguously indicates internal motions with correlation lengths of around 4.5 nm, that is, spanning over a large part of the molecule. We note that four out of the five simulations analyzed in Figure 5c display a weak shoulder at *q* ≈ 0.07 Å^−1^ resembling a rotation, which is a well‐understood artefact of cartesian coordinate principal component analysis (PCA) due to the mixing of rotational and internal dynamics.^[^ ^73^ ^]^

We remark that our analysis considers all dynamical contributions in one cumulant exponential term, so that no specific value of the internal relaxation time can be assigned to the slowest mode. However, the decays of the correlation functions (Figure 5a) occur on time scales between 10‐100 ns and the longer time scales correspond to smaller *q*, that is, longer distances. This provides a good estimate for the related relaxation time consistent with the previous nsFCS and MD simulation results.

As an independent validation of the absolute diffusion coefficients and thus the presented NSE analysis, we used neutron backscattering spectroscopy (NBS) which is expected to provide comparable diffusion coefficients in the high *q* limit of NSE. Following the conventional analysis (Figures S11 and S12, Supporting Information),^[^ ^50^ ^]^ we obtain information on both diffusion of the entire protein, and the geometrical confinement of small local motions down to sub‐nanosecond time scales. We obtain an apparent global diffusion coefficient of *D*_app_ = (3.27 ± 0.18) Å^2^ ns^−1^, consisting of the translational and rotational short‐time self diffusion coefficient,^[^ ^74^ ^]^ which is in excellent agreement with the NSE data. In addition, the confinement radius of local motions results in *a* = (1.95 ± 0.07) Å, which is fully consistent with the length of side chains limiting their motions. In particular, this finding stresses that local motions cannot explain the peak at *q* ≈ 0.13 Å^−1^, and larger concerted motions need to be at play.

### 2.5 The Nanosecond Dynamics of Hsp90 is State‐specific and Modulated by Sba1

**Figure 6:**
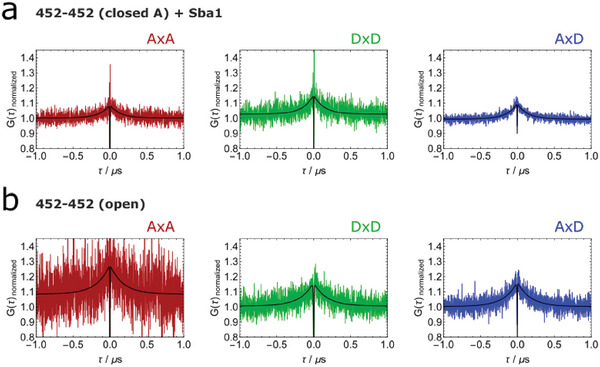
Modulation of Hsp90's nanosecond dynamics. Substate‐specific nsFCS analysis of the same construct as in Figure 2, namely the Hsp90 FRET pair 452‐452. a) Measurement in the presence of AMPPNP and Sba1 to further populate the closed state A and investigate the effect of Sba1. The bunching component is around (151  ±  14) ns, not significantly different from the value in absence of Sba1. b) Measurement in the absence of AMPPNP to populate the open state of Hsp90 and the analysis of this open state (*E* = 0.1–0.4). The bunching component is (187  ±  10) ns and therefore significantly different from the closed state A.

Next we test the effect of the addition of the co‐chaperone Sba1 in the presence of AMPPNP on the same Hsp90 construct as in Figure 2, labeled at position 452 (**Figure** **6**a). Sba1 was added without a label. Interestingly, we do not observe a significant shift in the bunching component of the closed A state. This at first sight surprising result can be explained by one known action of Sba1, namely the stabilization of the closed A state with respect to the open state, which likely does not include an overall change in the molecule‐spanning dynamics within this state. In other words, at position 452 still the same dynamic modes are most prevalent.

Until now, we have focused on the N‐terminal closed AMPPNP bound state of Hsp90. In the following we investigate if Hsp90's molecule‐spanning nanosecond dynamics is state‐specific. Therefore, we performed single‐molecule fluorescence experiments of Hsp90 in the absence of AMPPNP to further populate the N‐terminal open state and to analyze the nsFCS data for this state. We used the same variant (label at position 452) and dyes as shown in Figure 2. The nsFCS results are shown in Figure 6b. The data is again well described using one antibunching and one bunching components. Details on the fit results are shown in Table S1, Supporting Information. The bunching component is around 180 ns. Therefore, the molecule‐spanning dynamics of Hsp90 shifts from around 150 to 180 ns in between the N‐terminal closed and open state of Hsp90. This is significantly different with p < 0.05 given by Student's *t*‐test.

Finally, we tested the effect of the addition of Sba1 in the presence of AMPPNP to the Hsp90 construct labeled at position 61 in the N‐terminal domain (see Figure S3, Supporting Information). This position is at the binding site of Sba1^[^ ^24^ ^]^. Here, indeed a significant (p < 0.05) change of the dynamics is observed. This can be rationalized by a change in amplitude of some of the first eigenvectors upon binding of Sba1, which is in stunning agreement with MD simulations: The results from anisotropic network modeling (ANM) analysis for both Sba1‐bound and unbound Hsp90 are displayed in Figure S13, Supporting Information. In ANM, small eigenvalues approximately indicate slow normal mode frequencies. Similar to the results from the cartesian PCA, slow modes represent global motions of the full dimer. The eigenvalue distribution itself does not exhibit any difference between the two states, so the frequency distribution of Hsp90 appears to remain unchanged upon Sba1 binding in this simple model. However, individual normal modes exhibit locally changed dynamics: Eigenmode 4 is the slowest mode predicted with a change in dynamics around the binding site of Sba1. For comparison, we show eigenmode 2, which represents a similar twist motion, but without effect of Sba1. Sba1 therefore likely locally dampens molecule‐spanning motions of the dimer, which is in agreement with our results from nsFCS experiments.

## 3 Conclusion

Combining advanced fluorescence techniques, quasi‐elastic neutron scattering and full‐atom MD simulations, we shed light on the previously unaddressed nanosecond dynamics of the molecular chaperone Hsp90 (see Figure 1). The complementary use of these methods rooted in bio‐ and soft matter physics offers unique insights because temporal and spatial information is obtained simultaneously in a coupled manner. Furthermore, the specificity of the techniques employed allowed us to distinguish internal dynamics from global rotation, although they occur at similar time scales.

**Figure 7:**
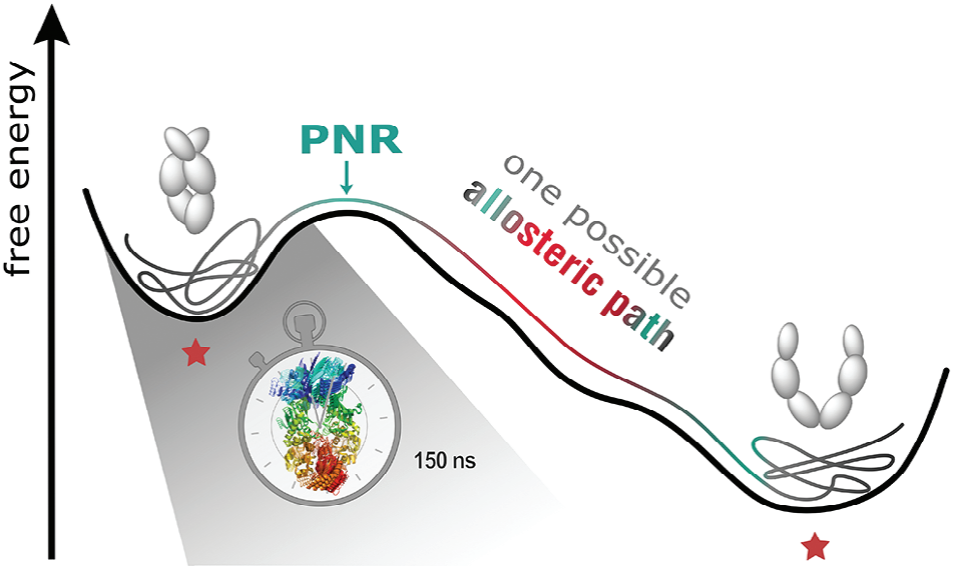
Molecule‐spanning dynamics on the 150 ns time scale might be precursors of large conformational changes between two equilibrium states (marked with red stars). They probe higher regions of the free energy landscape and likely guide the protein toward the point of no return (PNR) to complete, for example, an allosteric signaling path. Only one exemplary path, which might result from the highly diffusive motions, is depicted here.

Most interestingly, we observe molecule‐spanning dynamics that are distributed across the complete protein on the 100 to 200 ns time scale. This internal dynamics can be described as a diffusive motion, in the present case a bending contraction along the N‐M axis of the closed Hsp90 dimer. This motion cannot be described by simple harmonic oscillations anymore. Usually a simple oscillatory motion such as a normal mode is used for protein dynamics, which then defines in a Kramer's picture an attempt rate (sometimes also referred to by attempt frequency) without damping.^[^ ^75^ ^]^ Instead, we show that we have highly diffusive dynamics along different eigen vectors. Interestingly, the 150 ns timescale is on the order of magnitude as the Kramer's attempt rate for proteins is estimated.^[^ ^76^ ^]^ Therefore, we are convinced that we do not only probe the minimum of the free energy of the closed and open state of Hsp90, but already explore higher regions of the free energy landscape. Slow nanosecond motions are likely precursors of larger conformational changes and those in this work identified might be the ones that indeed lead to the larger conformational changes depicted in **Figure** **7**. The respective free energy barriers probed by these dynamics may for example, lead to the formation of a more contracted protein state upon ATP hydrolysis and thus belong to an allosteric path connecting the nucleotide binding site and the full protein.^[^ ^56^ ^]^

We further showed that these nanosecond‐range dynamics of Hsp90 are state‐specific, that is, they are different in Hsp90's N‐terminal open state compared to the closed state. In addition they can be affected by a co‐chaperone in a position‐dependent way. If and how these observations might be functionally relevant has to be seen in the future. Also if the molecule‐spanning dynamics are a basis for structural allostery, that is, changing the structure of a protein at a distant site, or dynamic allostery,^[^ ^77^, ^78^, ^79^ ^]^ where protein functional changes are mediated by changing the dynamics of a protein. Slowing down the dynamics of Hsp90 could facilitate its interaction with clients by populating the binding‐competent state for longer times and therefore making it more accessible. Altogether, our integrative study shows molecule‐spanning dynamics on the 150 ns time scale for the Hsp90 protein. This dynamic depends on the conformational state of Hsp90. It might be a precursor for large conformational changes, as it does not only probe the free energy minimum and could therefore be relevant for protein regulation.

## 4 Experimental Section

### Protein Production and Purification

Gene expression and subsequent protein purification were performed as previously described.^[^ ^80^ ^]^ Yeast Hsp90 wildtype and variants with cysteine mutations at positions 61 and 452 for fluorescent labeling were recombinantly produced in *E. coli*. Protein purification was performed with three consecutive chromatography steps, Ni‐affinity, anion‐exchange, and size exclusion chromatography, yielding nearly monodisperse dimeric Hsp90. Yeast Sba1 was produced and purified accordingly. Altogether about 1 g of protein was purified for all the experiments. A detailed description is given in the Section S3.1, Supporting Information.

### Fluorescent Labeling and Single‐Molecule Experiments

Fluorescent labeling was achieved by site‐directed cysteine‐maleimide chemistry. The maleimide derivate of Atto550 was used as the donor and Atto647N as the acceptor, respectively (ATTO‐TEC GmbH). Note that donor and acceptor positions of a FRET pair were always specified in the order “donor–acceptor”. To stabilize the Hsp90 dimer at single‐molecule concentration a coiled‐coil motif (DmKHC, *D. melanogaster*) was inserted at the C‐terminus which prevented dimer dissociation. Hsp90 heterodimers with one donor and one acceptor molecule per dimer were obtained by incubating donor‐ and acceptor‐labeled Hsp90 dimers for 45 min at 43 °C at ratio 1:1. At this temperature monomers exchanged which yielded stochastically one half Hsp90 heterodimers. The samples were centrifuged for 1 h at 4 °C to separate potential aggregates. As buffer 40 mm HEPES, 150 mm KCl, and 10 mm MgCl_2_ were dissolved in ultra pure H_2_O and pH was adjusted to 7.4–7.5. Final protein concentrations were ≈200 pm for single‐molecule and ≈ 200–1000 pm for subpopulation‐specific nsFCS experiments. AMPPNP was added to the samples immediately before the start of the measurement such that the final nucleotide concentration was 2 mm. Measurements were performed with 0.1025 mg mL^−1^ bovine serum albumin (BSA). Sba1 was added such that the final co‐chaperone concentration was ≈10 µm which is above the Sba1‐Hsp90 dissociation constant of 1.75 µm.^[^ ^81^ ^]^

### Sample Preparation for Neutron Scattering Experiments

In all neutron experiments the yeast Hsp90 wildtype construct without the coiled‐coil motif and without cysteine mutation was measured. A buffer exchange from H_2_O‐based buffer to 150 mm KCl and 10 mm MgCl_2_ dissolved in D_2_O was achieved by five consecutive dialysis steps at 8 °C over night (Slide‐A‐Lyzer 10 K, Thermo Scientific). To ensure that the signal of free H_2_O was reduced sufficiently, a D_2_O‐based buffer excess of >50:1 was applied in each dialysis step. Directly before the measurement samples were centrifuged 20 min at 12,857 g and 8 °C. Final protein concentrations determined by UV–vis spectroscopy (Nanodrop ND‐1000, Thermo Fisher Scientific) were 614 µm.

### Check of Bio‐Functionality

Bio‐functionality of the Hsp90 dimer was controlled according to well‐established ATPase assays.^[^ ^82^ ^]^ The assay couples ATP hydrolyzation to oxidation of NADH to colorless NAD+ which was monitored over time on a Lambda35 UV–vis spectrometer. After the addition of 2 mm ATP and 2 µm Hsp90 a linear decrease of the absorbance at 340 nm was obtained (see Figure S10b, Supporting Information). Figure S10c, Supporting Information, shows the measured ATPase rates. In addition, the open‐close dynamics of Hsp90 in D_2_O‐based buffer by single‐molecule experiments (see Figure S14, Supporting Information) were validated.

### Fluorescence Experiments

Single‐molecule fluorescence experiments were conducted on a homebuilt confocal setup as depicted in Figure S2, Supporting Information at 295 K. Green (532 nm, LDH‐D‐FA‐530L, PicoQuant) and/or red laser light (640 nm, LDH‐D‐C‐640, PicoQuant) were used to excite donor and acceptor dyes. Before focusing on the sample by a 60× water immersion objective (CFI Plan Apo VC 60XC/1.2 WI, Nikon), both beams were polarized and overlaid by a dichroic mirror (zt 532 RDC, AHF). In the emission path a second dichroic mirror (F53‐534 Dual Line beam splitter z 532/633, AHF) separated donor fluorescence from acceptor fluorescence. Pinholes (100 µm diameter, if not described otherwise) filtered off‐focus light. Before detection, polarizing beam splitters separated parallel and perpendicular polarized light. Donor and acceptor emission was detected by single‐photon detectors (two SPCM‐AQRH‐14, Excelitas and two PDM series APDs, Micro Photon Devices).

### Passivation and Chamber Assembly

For the fluorescence experiments SiPEG surface‐passivated chambers were used. Therefore high precision coverslips (Carl Roth, 24 × 60 mm, 170 ± 5 µm) were cleaned by sonicating in 2% Hellmanex (Hellma Analytics), ultra pure water and isopropanol:water mixture (≈1:3) for dissolving impurities. The remaining droplets were removed with nitrogen. The activation of the slides was done by low pressure plasma (Tetra 30, diener electronics) performing subsequently an O_2_ and air plasma process. The plasma process was directly followed by treating the cover glasses with methoxy‐silane‐polyethylenglycol (mSiPEG) solution (5 kDa, Rapp Polymere GmbH) with a concentration of 30 mg mL^−1^. Unbound mSiPEG was washed away with pure water. For long‐time measurements of more than 1 h, the passivated coverslips were used to build a ≈200 µL cavity by a two‐component silicon polymer (Microset 101, Microset Products Ltd). To prevent evaporation another passivated coverslip was used as a lid “glued” with Korasilon Paste mittelviskos (Kurt Obermeier GmbH & Co. KG). The chamber was pre‐incubated with BSA (0.5 mg/mL) for ≈20 min.

### Subpopulation‐Specific (nanosecond) FCS

Green or red cw laser excitation was used to generate nsFCS data. The laser power (green cw) was 475 µW for nsFCS data of Atto550‐ and Atto647N‐labeled Hsp90. Additional IR filter (LC‐HSP750‐25, LaserComponents) were inserted before the detectors to reduce artefacts from detector afterglowing.^[^ ^83^ ^]^ Photon arrival times were saved in T2 mode (HydraHarp400, PicoQuant) which provided a time‐resolution of 1 ps. nsFCS data analysis was performed using the Wolfram Mathematica package Fretica (https://schuler.bioc.uzh.ch/programs).^[^ ^40^ ^]^ Single‐molecule events were identified using the Δ*T* burst search algorithm with the used parameters given in Table S1, Supporting Information. The number of photons is corrected by the “route correction matrix (RCM).” Here, the correction parameters were determined by a smPIE experiment. Subpopulation‐specific analysis was achieved based on the FRET efficiency *E* according to Equation (4)

(4) $$E=\frac{GF_{\text{DA}}-\alpha F_{\text{DD}}-\delta F_{\text{AA}}}{\gamma F_{\text{DD}}+F_{\text{DA}}-\alpha F_{\text{DD}}-\delta F_{\text{AA}}}$$

Here, *F*_*ij*_ stands for the detected fluorescence intensities which are detected in channel *i* after excitation of *j* (*i*, *j* = {D, A}, with D = donor, A = acceptor), respectively. *E* was corrected for crosstalk by α, for direct excitation by δ and for local differences in quantum yield and detection efficiency by γ. The G‐factor corrected for differences between parallel and perpendicular detection channels. The substate‐specific nsFCS curves of the open and closed A state were obtained by auto‐ and cross‐correlating single‐molecule events corresponding to FRET efficiencies in the range from 0.1–0.4 and 0.4–0.8, respectively.

Auto‐ and cross‐correlations were calculated for donor–donor (D×D), acceptor–acceptor (A×A) and acceptor–donor emission signals (A×D), respectively. All nsFCS experiments were measured with continuous green excitation. For D×D and A×A parallel and perpendicular channel signals were cross‐correlated to avoid artefacts resulting from afterpulsing. Logarithmic binned FCS curves were calculated from picoseconds to seconds, while linear binned correlations for nsFCS zoomed in the time window of 1000 ns with a lag time of 1 ns. This part was described by Equation (5).

(5) $$G\left(\tau \right)=a\left(1-c_{\text{ab}}e^{-|\tau -\tau_{0}|/\tau_{\text{ab}}}\right)\left(1+c_{b}e^{-|\tau -\tau_{0}|/\tau_{b}}\right)$$

The fit models includes a scaling factor *a*, the weight of the antibunching mode *c*_ab_, the weight of the bunching mode *c*_b_, the photon antibunching time τ_ab_, and the bunching time τ_b_. τ_0_ corrects for a small delay in the detection channels. Note that τ_b_ is a global fit parameter which was used to describe D×D, A×A, and A×D correlation data. A summary of all fit results is given in Table S1, Supporting Information. The corresponding logarithmic FCS curves were fitted using Equation (6).

(6) $$G\left(\tau \right)=\frac{\left(1-c_{\text{ab}}e^{-\tau /\tau_{\text{ab}}}\right)\left(1+c_{b}e^{-\tau /\tau_{b}}\right)\left(1+c_{T}e^{-\tau /\tau_{T}}\right)}{n*\left(1+\frac{\tau }{\tau_{D}}\right)\sqrt{1+\frac{\tau }{\kappa^{2}\tau_{D}}}}$$

The indices of the weights *c*_*i*_ and of the lag times τ_*i*_, namely “ab”, “b”, “T” and “D”, refer to antibunching, bunching, triplet kinetics, and diffusion dynamics, respectively. *n* is a scaling factor, containing the average intensity, the average background signal, and the true number of molecules in the confocal volume. κ describes the eccentricity of a confocal volume, assuming 3D Gaussian shape. The lag times and κ were globally fitted with τ_b_ and τ_T_ fixed to beforehand determined values. Note that for the cross‐correlation of the signal the triplet term was excluded.

### Time‐Resolved Anisotropy

To obtain time‐resolved single‐molecule anisotropy decays pulsed‐interleaved excitation was used to alternately excite donor and acceptor molecules at 20 MHz repetition rate. Excitation powers directly before the objective were 317 and 114 µW for green and red, respectively. Microtimes and macrotimes were recorded in T3 mode by time‐correlated single photon counting (HydraHarp400, PicoQuant) with 50 ns and 16 ps time‐resolution, respectively. Data were analyzed in Wolfram Mathematica with the package Fretica (main text and Figure 3), and in Matlab by the software PAM^[^ ^84^ ^]^ (Figure S5, Supporting Information). The corresponding analysis with PAM is described in Section S3.3, Supporting Information. Single‐molecule events of 68 consecutive 1‐h measurements were identified by the Δ*T* burst search algorithm combined with dual channel burst search (Fretica) with the following parameters set: Δ*T* = 100µs, *N*_min_ = 35, and *N*_max_ = 10 000. For each single‐molecule event FRET efficiency *E*, stoichiometry *S*, and anisotropy *r* were calculated according to Equations (4), (7), and (8), respectively.

(7) $$S=\frac{\gamma F_{\text{DD}}+F_{\text{DA}}-\alpha F_{\text{DD}}-\delta F_{\text{AA}}}{\gamma F_{\text{DD}}+F_{\text{DA}}-\alpha F_{\text{DD}}-\delta F_{\text{AA}}+\beta F_{\text{AA}}}$$

In addition to the correction factors α, δ, and γ, the stoichiometry is corrected by β to account for differences in absorption cross‐section and excitation powers of donor and acceptor fluorophores, respectively.

(8) $$r=\frac{GF_{∥}-F_{⊥}}{\left(1-3l_{2}\right)GF_{∥}+\left(2-3l_{1}\right)F_{⊥}}$$

Here, subscripts of the fluorescence intensities *F* denote the polarization channel. *l*_1_ and *l*_2_ account for the depolarization effect of the objective and were set to *l*_1_ = 0.0308 and *l*_2_ = 0.0368.^[^ ^85^ ^]^ *E* and *S* were used to filter and histogram microtimes belonging to a specific conformational state. The subpopulation‐specific acceptor anisotropy decays were analyzed with Fretica using the *cone‐in‐cone* model:^[^ ^47^ ^]^

(9) $$\begin{array}{ccc} r(t) & = & r_{0}\left(\left(1-A_{\mathrm{dye}}\right)e^{-t/\rho_{\mathrm{dye}}}+A_{\mathrm{dye}}\right)(\left(1-A_{\mathrm{local}}\right)e^{-t/\rho_{\mathrm{local}}}\\ & & +A_{\mathrm{local}})e^{-t/\rho_{\mathrm{global}}} \end{array}$$

Here, ρ_dye_ accounts for correlations due to dye self‐rotation, ρ_local_ for rotational dynamics of local structural elements the dye is attached to, and ρ_global_ for rotation of the overall protein. The global rotation of the protein and the self‐rotation of the dye was globally fitted over all three separated FRET‐populations and *r*_0_ was fixed to 0.4. Note that a reconvolution analysis was not necessary because the time scale of interest (>1 ns) was well separated from the time scale of instrument response (compare Figure S4, Supporting Information). As a control, other anisotropy fit models were tested in PAM with its established fit models including 1 to 3 rotational components and reconvolution analysis (Figure S5 and Table S6, Supporting Information).

### Computational Methods

MD simulations were prolongation runs of the 5 ×  1 µs simulation runs of a AMPPNP‐bound Hsp90 dimer in the closed conformation presented in ref. [56]. In brief, simulation systems were generated from PDB ID 2CG9^[^ ^24^ ^]^ and simulated using Gromacs v2020^[^ ^86^ ^]^ with the Amber99SB*ILDN‐parmbsc0‐χ *OL*3 + AMBER99ATP/ADP force field^[^ ^87^ ^]^ with the TIP3P water model^[^ ^88^ ^]^ in an NPT ensemble at 1 bar and 310 K. The respective simulations were continued by a further 5 ×  1 µs and treated the initial 1 µs from an earlier work as an equilibration period. Coordinates were saved each 100 ps.

Accessible dye volumes (AVs) were calculated based on structural MD snapshots using the Olga software.^[^ ^64^, ^89^ ^]^ Before both, the analysis of accessible volumes and the principal component analysis of backbone dynamics, contributions from translational and rotational diffusion to the overall dynamics were removed via a translational and rotational fit of each trajectory snapshot to the protein structure after initial equilibration. As a fit reference, all alpha‐helix and beta‐sheet backbone atoms were used as identified via DSSP in Pymol. AVs were calculated based on the equilibrated 5 × 1 µs MD traces of the Hsp90‐AMPPNP simulations. This resulted in 10 000 accessible volumes with a time resolution of 100 ps averaged over five independent MD runs.

Translational diffusion coefficients and principal axes of inertia were calculated with Gromacs‐internal tools. The rotation autocorrelation function *G* ^rot^(τ) was calculated from the first principal axis of the moment of inertia's **I** cartesian *i* = *x*, *y*, *z* components *I*_*i*_

(10) $$\begin{array}{c} G^{\mathrm{rot}}\left(\tau \right)=\frac{1}{3N}\sum_{i=x,y,z}^{i}\sum_{j}^{N}\frac{\left(I_{i}\left(t_{j}+\tau \right)-\langle I_{i}\rangle \right)\left(I_{i}\left(t_{j}\right)-\langle I_{i}\rangle \right)}{\sigma_{I_{i}}^{2}} \end{array}$$

for all *N* discrete time steps *t*_*j*_ that can be overlapped with the original time series within a shift τ, time series means <*I*_*i*_(*t*_*j*_) >, and variance $\sigma_{I_{i}}^{2}$. To remove artifacts in the calculation *G* ^rot^(τ) owing to random sign flips in the **I** time series, the sign of **I** was flipped in case any *I*_*i*_ changed by more than 0.5 units between two time steps.

The cartesian principal component analysis (cPCA)^[^ ^90^ ^]^ was carried out using Gromacs‐internal tools independently for all five AMPPNP trajectories. The charged loop between residue numbers 208 and 280 was excluded from analysis due to its high flexibility and according to dominance of the first principal components. After a rotational and translational fit of the protein C_α_ atoms to the final protein structure, the mass‐weighed cartesian covariance matrix of the protein backbone and adjacent C_β_ atoms was calculated and diagonalized. The resulting eigenvectors and eigenvalues were subsequently used in the analysis of neutron scattering experiments.

The anisotropic network model (ANM) analysis was performed using the ANM web server v2.1.^[^ ^91^ ^]^ As in simulations, PDB ID 2CG9 was used as input. For analysis of the effect of the Sba1 co‐chaperone on the Hsp90 dimer, one structure was created with only one Sba1 unit, and one without any co‐chaperone. As the crystal structure contained truncated loop domains without any connection to the protein bulk, which caused the appearance of artificial localized normal modes, amino acids 208 to 267 were removed from the Hsp90 dimer, and amino acids 130 to 135 from Sba1.

Simulation data was analyzed using Numpy,^[^ ^92^ ^]^ Scipy,^[^ ^93^ ^]^ and pandas^[^ ^94^ ^]^ libraries. Structural data was visualized using Pymol^[^ ^95^ ^]^ and VMD.^[^ ^96^ ^]^

### Neutron Scattering Experiments

Neutron scattering offers non‐destructive access to the nano‐scale. Different techniques provided access to the structural and dynamical properties of the investigated samples at different time scales reaching from dynamics on the pico‐second time scale to static structures.^[^ ^50^ ^]^ The coherent and incoherent isotope‐dependent scattering cross‐sections led to the coherent and incoherent scattering signal, which summed up the total scattering signal. The fraction of the different contributions depended on the investigated scattering angle 2θ which together with the wavelength λ could be linked to the momentum transfer  ℏ**q** whereby *q* = |**q**| is the absolute value of the scattering vector

(11) $$q=\frac{4\pi \sin\left(\frac{2\theta }{2}\right)}{\lambda }$$

### Neutron Spin Echo Spectroscopy

NSE experiments were performed at the spin‐echo spectrometer IN15 (ILL, Grenoble).^[^ ^97^ ^]^ Samples were measured at 50 mg mL^−1^ in quartz cuvettes (2 mm thickness) at 295 K. Four different detector angles were configured with different wavelengths (2Θ  =  8.3°, λ  = 6 Å ; 2Θ  =  3.5°, λ  = 10 Å ; 2Θ  =  6.5°, λ  =  10 Å ; 2Θ  =  9.5°, λ  = 10 Å). The position sensitive detector was subdivided into three *q* values, resulting in twelve *q*‐values between 0.028 Å ;^−1^ < *q* < 0.16 Å ;^−1^. Standard methods were applied for data reduction and background subtraction as detailed in refs. [98, 99].

### Quasi‐Elastic Neutron Backscattering

NBS data were measured at the backscattering spectrometer IN16B (ILL, Grenoble) at 295 K. The sample measured by NSE and NBS was identical which ensured the best data consistency. To this end, the 50 mg mL^−1^ Hsp90 solution was transferred from the NSE quartz cuvette to a cylindrical aluminum holder with a 0.15 mm gap between the inner and outer radius. Unpolished Si(111) analyzers were used in combination with a chopper ratio 1:1 (“high flux” mode) and with the Doppler monochromator. D_2_O buffer and vanadium were measured for 4 h to correct for background signal and to determine the energy resolution, respectively.

### Statistical Analysis

nsFCS data: Correlations were calculated based on the algorithm by Laurence.^[^ ^100^ ^]^ Single‐molecule events were identified by the Δ*T* method. For reasons of direct comparability, the data were normalized by the amplitude *a* which was obtained as a fit result.

Time‐resolved Anisotropy: TRA data were processed in Fretica by using the Δ*T* burst search algorithm combined with dual channel burst search. In PAM, the TRA data was analyzed using an all‐photon burst search algorithm with a count rate threshold criterion.^[^ ^84^ ^]^ In detail, the “Sliding Time Window method”^[^ ^84^ ^]^ was used to identify single‐molecule events.

Neutron spectroscopy: Standard methods were applied for data reduction: The NBS data were reduced using Mantid (www.mantidproject.org): Normalization to the incident beam monitor, and subtraction of the container signal. All reductions were traceable in the reduced hdf files equally curated by the ILL and available by ref. [101]. NSE: NSE data were reduced using in‐house routines written in Igor Pro. Graphite was used to calibrate the resolution and the transmission weighted signal from the buffer was subtracted from all samples. The counting time was on the order of 4 h for each sample and temperature in a neutron spectroscopy experiment (backscattering and spin‐echo). Error bars per point were calculated based on Poisson counting statistics and propagated via standard error propagation throughout data reduction.

For the neutron scattering data, the well‐established chi‐squared test was employed to evaluate the goodness of the fit achieved by a non‐linear least‐squares minimization algorithm (e.g., MATLAB lsqcurvefit).

MD simulations: Simulations were performed in the form of five statistically independent trajectories (see ref. [56]). Data evaluation was performed on all trajectories independently. Errors were then calculated as the standard error of the mean for the *N* =5 independent simulations.

Uncertainties: +/− values are standard deviations between independent repeats, if not stated otherwise. All standard fit errors are given in the tables in the Supporting Information.

## Conflict of Interest

The authors declare that there is no conflict of interest.

## Author Contributions

B.S., C.B., and V.F. contributed equally to this work. B.S., C.B., F.R., F.S., T.S., and T.H. designed the research; B.S., C.B., V.F., M.G., T.S, and I.H. performed the measurements; S.W. performed MD simulations; B.H., V.F., C.G., and B.S. prepared the samples. All authors contributed to the data analysis and interpretation and all authors wrote the manuscript.

## Supporting information

Supporting Information

Supplemental Movie 1

Supplemental Movie 2

Supplemental Movie 3

Supplemental Movie 4

## Data Availability

Neutron data from IN15, IN16b and D11 as well as the fluorescence data are associated with the neutron beamtime proposal 8‐04‐838.^[^ ^101^ ^]^
