# Supplementary material for: The Onset of Molecule‐Spanning Dynamics in Heat Shock Protein Hsp90
Source: Adv Sci (Weinh). 2023 Nov 20;10(36):2304262. doi: 10.1002/advs.202304262 (PMC10754087; doi:10.1002/advs.202304262)
Supplement: Supplementary file 1 — Supporting Information [file ADVS-10-2304262-s001.pdf]

ADVANCED SCIENCE
Open Access

Supporting Information

for *Adv. Sci.*, DOI 10.1002/advs.202304262

The Onset of Molecule-Spanning Dynamics in Heat Shock Protein Hsp90

Benedikt Sohmen, Christian Beck, Veronika Frank, Tilo Seydel, Ingo Hoffmann, Bianca Hermann, Mark Nüesch, Marco Grimaldo, Frank Schreiber, Steffen Wolf*, Felix Roosen-Runge* and Thorsten Hugel**

Supporting Information: The onset of molecule-spanning dynamics in heat shock protein Hsp90

Benedikt Sohmen[‡1], Christian Beck[‡2,3], Veronika Frank[‡1], Tilo Seydel[3], Ingo Hoffmann[3], Bianca Hermann[1], Mark Nüesch[4], Marco Grimaldo[3], Frank Schreiber[*2], Steffen Wolf[*5], Felix Roosen-Runge[*6], and Thorsten Hugel[*1,7]

[1]Institute of Physical Chemistry, University of Freiburg, Albertstrasse 21, 79104 Freiburg, Germany
[2]Institute of Applied Physics, University of Tübingen, Auf der Morgenstelle 10, 72076 Tübingen, Germany
[3]Institut Max von Laue - Paul Langevin, 71 avenue des Martyrs, 38042 Grenoble, France
[4]Department of Biochemistry, University of Zurich, Winterthurerstrasse 190, CH-8057 Zurich, Switzerland
[5]Biomolecular Dynamics, Institute of Physics, University of Freiburg, Hermann-Herder-Strasse 3, 79104 Freiburg, Germany
[6]Department of Biomedical Sciences and Biofilms-Research Center for Biointerfaces (BRCB), Malmö University, 20506 Malmö, Sweden
[7]Signalling Research Centers BIOSS and CIBSS, University of Freiburg, Schänzlestrasse 18, 79104 Freiburg, Germany
[‡]These authors contributed equally
[*]contact details: frank.schreiber@uni-tuebingen.de; steffen.wolf@physik.uni-freiburg.de; felix.roosen-runge@mau.se; thorsten.hugel@physchem.uni-freiburg.de

1 Supplementary Figures

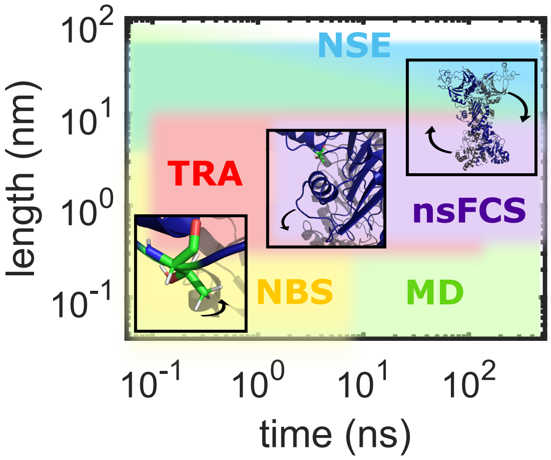

Figure S1: Overview of employed techniques. Nanosecond fluorescence correlation spectroscopy (nsFCS), time-resovled anisotropy (TRA), neutron spin echo (NSE), neutron backscattering (NBS) and full-atom molecular dynamics (MD) simulations cover dynamics from ps to µs and length scales from sub-nm to ∼100 nm.

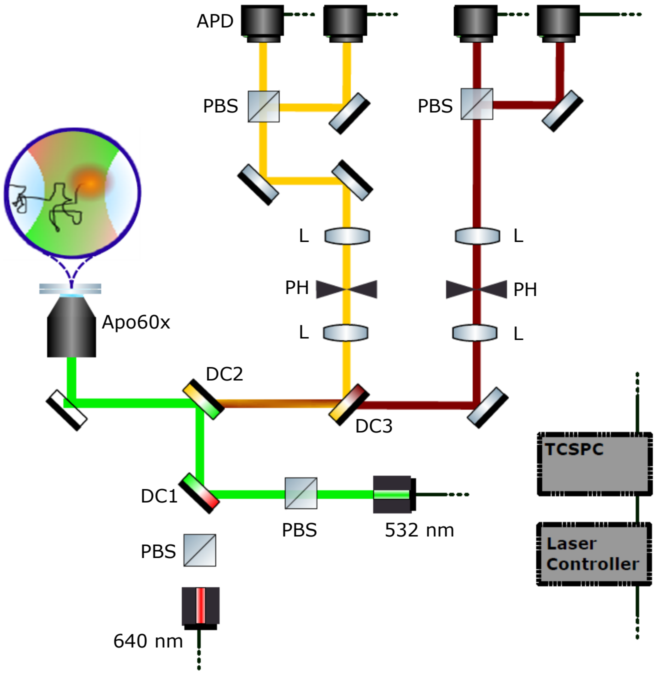

Figure S2: Scheme of the confocal single-molecule fluorescence set-up, here operated in continuous wave (cw) excitation mode. Depending on the type of experiment, dyes were excited with 532 nm (cw) or 532 nm and 640 nm (pulsed-interleaved). Polarizing beam splitters (PBS) were used to generate polarized excitation light and polarization-sensitive detection. Spectral overlay and separation was achieved by dichroic mirrors (DC): overlay of green and red excitation pulses (DC1), separation of excitation light, scattered light and fluorescence emission (DC2) and separation of donor- and FRET-based acceptor emission (DC3). Light was focussed and re-collected by an apochromat (Apo 60x). Lenses (L) in combination with pinholes (PH) were used to achieve confocal single-molecule detection. Single photon detection with picosecond time-resolution was achieved by avalanche photon diodes (APD) and processed by a time-correlated single photon counting (TCSPC) unit. APDs, TCSPC unit, laser controller and lasers are connected as indicated by dotted black lines. Scattered laser light was in addition blocked by a notch filter in each of the detection paths.

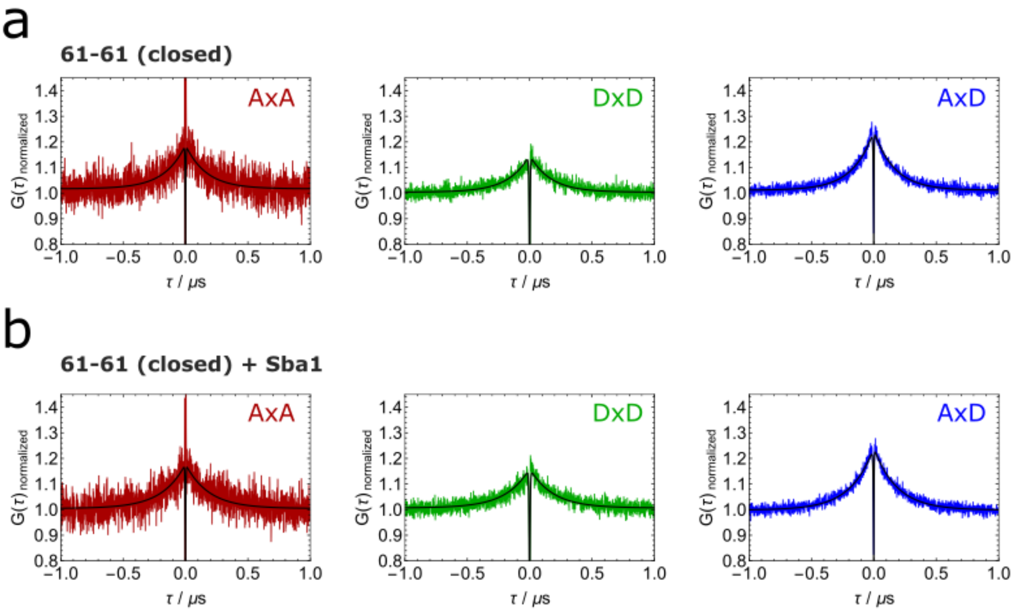

Figure S3: Substate-specific nsFCS data and fits of Hsp90 FRET pair 61-61 in presence of AMPPNP with (Fig. S3b) and without Sba1 (Fig. S3a). Closed-state dynamics of Hsp90 were analyzed by selecting only single-molecule events belonging to the closed AMPPNP state (E=0.1-0.3). For each sample, A×A (red), D×D (green) and A×D (blue) linear binned correlations are shown. Each data set is described by an individual antibunching time ($\sim$3 ns) and a global bunching time of (165 ± 4) ns in the absence of Sba1 and (188 ± 2) ns in the presence of Sba1. All fit parameters are summarised in Tab. S1.

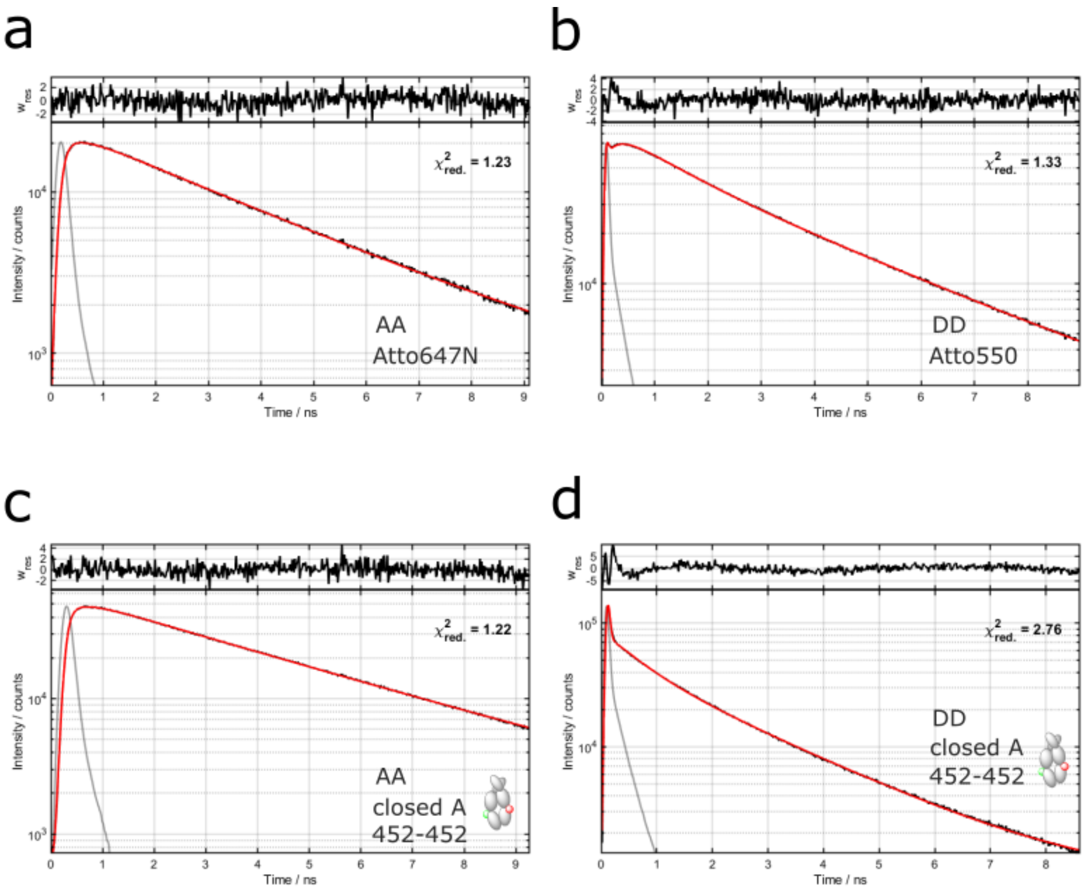

Figure S4: Lifetime analysis of free dye in solution and Hsp90 with the labeling position 452-452 by PAM established lifetime fitmodels with reconvolution of instrument response function IRF: a) analysis of TCSPC histograms of the Maleimide derivate of Atto550 in pure water and b) of the Maleimide derivate of Atto647N in pure water. c) Analysis of TCSPC histograms of the acceptor channel AA for Hsp90's closed A conformation. d) Analysis of TCSPC histograms of the donor channel DD for Hsp90's closed A conformation. For details of the analysis please refer to the method section. Tab. S5 contains the fit results.

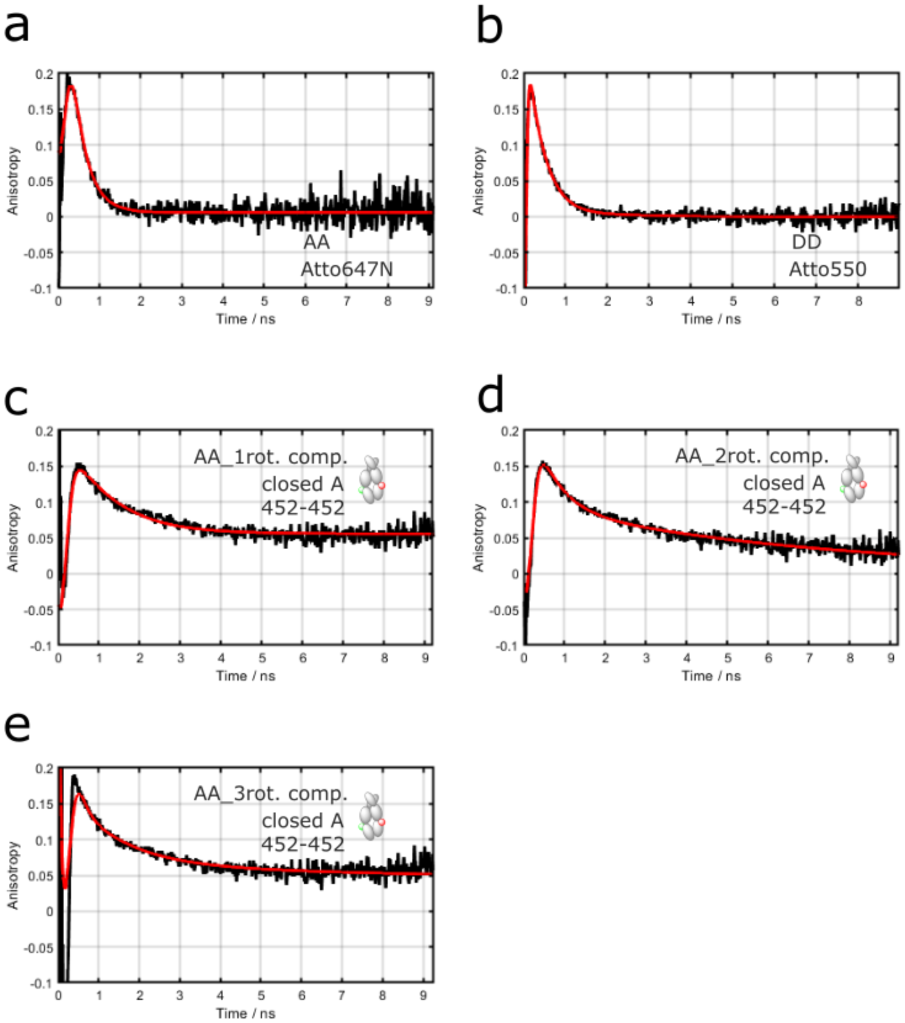

Figure S5: Time-resolved single-molecule anisotropy decays. The data was analyzed by PAM with its integreted fit models which is denoted in brackets. a) TRA decay of the maleimide derivate Atto647N with 1 rotational component and 1 lifetime ('Fit Anisotropy'). b) TRA for the maleimide derivate of Atto550 with 1 rotational components and 2 lifetimes ('Fit Anisotropy (2 exp lifetime)'). c)-e) The FRET pair 452-452 was labeled with Atto550 and Atto647N and measured in presence of AMPPNP at 22°C. FRET efficiency vs. stoichiometry analysis was applied to identify and select the conformational sub-state 'closed A' of Hsp90. For the data processing, 47 consecutive 1-hour measurements were combined. The acceptor anisotropy decay after acceptor excitation for closed state A was fit by a model including c) 1 rotational component ('Fit Anisotropy'), d) 2 rotational components ('Fit Anisotropy (2 exp rot') and e) 3 rotational components ('Fit Anisotropy (4 exp lifetime, 4 exp rot)' - here the fit was modified by specifying the fit range for three rotational parameters and one lifetime parameter and setting the other parameters to zero after global fitting of the TCSPC histograms for parallel and perpendicular detection channel, followed by reconvolution. The fit results are shown in Tab.S6.

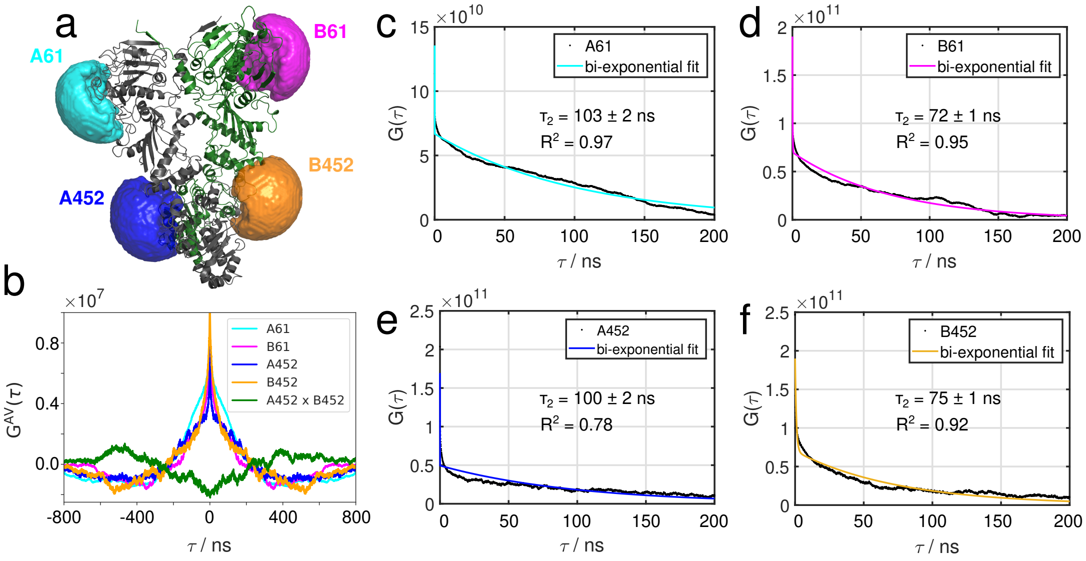

Figure S6: MD-based accessible dye volume correlation analysis for Hsp90 with two AMPPNP molecules. a) Structural MD snapshot of the Hsp90 dimer with accessible dye volumes at positions 61 and 452 at chain A and/or B, respectively. b) Accessible dye volume auto- and cross-correlations. c)-f) Accessible dye volume autocorrelations with unconstrained bi-exponential fits at respective positions in the Hsp90 dimer. Correlations at 70-100 ns are observed which hint towards local structural dynamics affecting the accessible dye volumes. See Tab.S3 for all fit results.

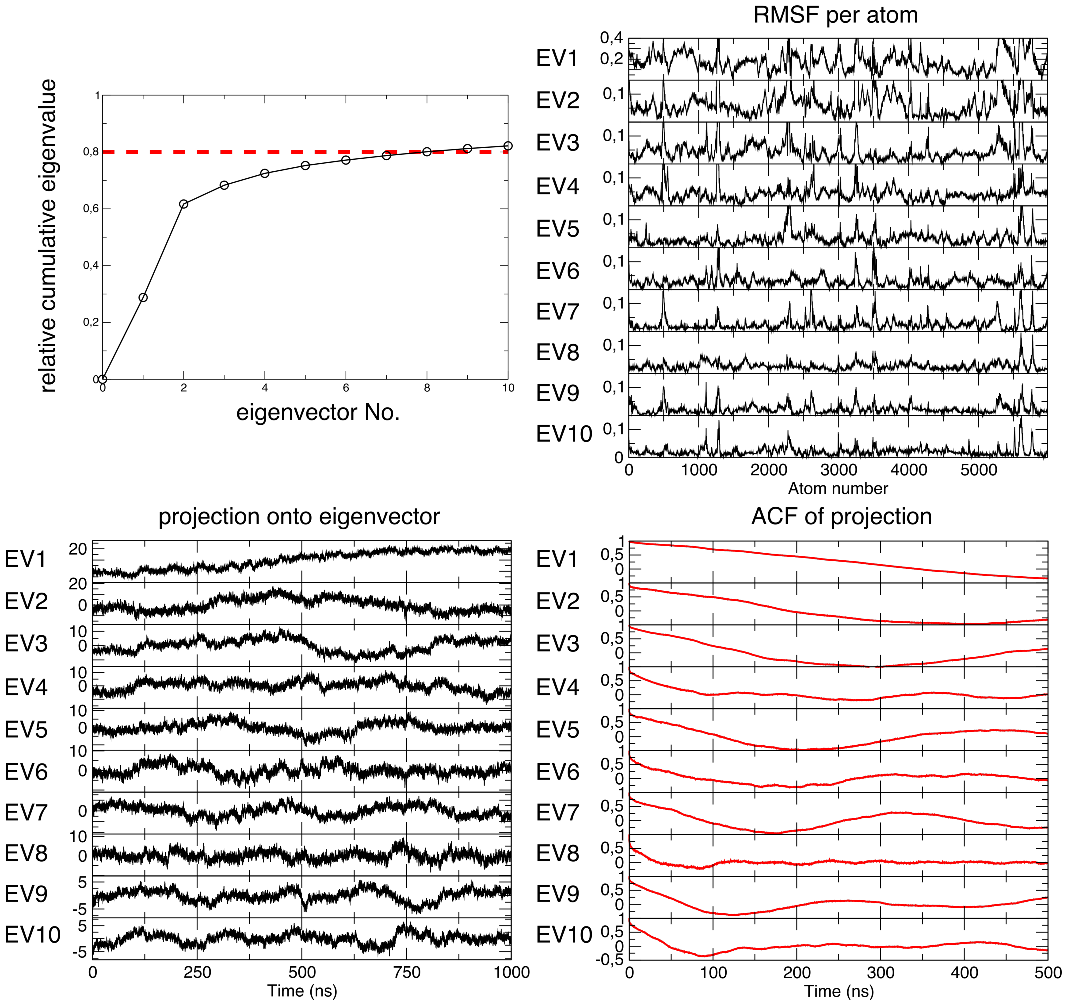

Figure S7: Results from cartesian PCA of one exemplary 1 μs simulation. Top left: Cumulative relative eigenvalues. While eigenvectors 1 and 2 contain the majority of variance, eight eigenvectors need to be considered to cover 80% of the closed dimer's dynamics. Top right: root mean square fluctuations of individual atoms covered in the first ten eigenvectors. All ten vectors represent global motions involving the full dimer. Bottom: projections of one exemplary trajectory onto eigenvectors and their ACF. While all projections seem to contain slow oscillations, they are overlaid by fluctuations on short time scales.

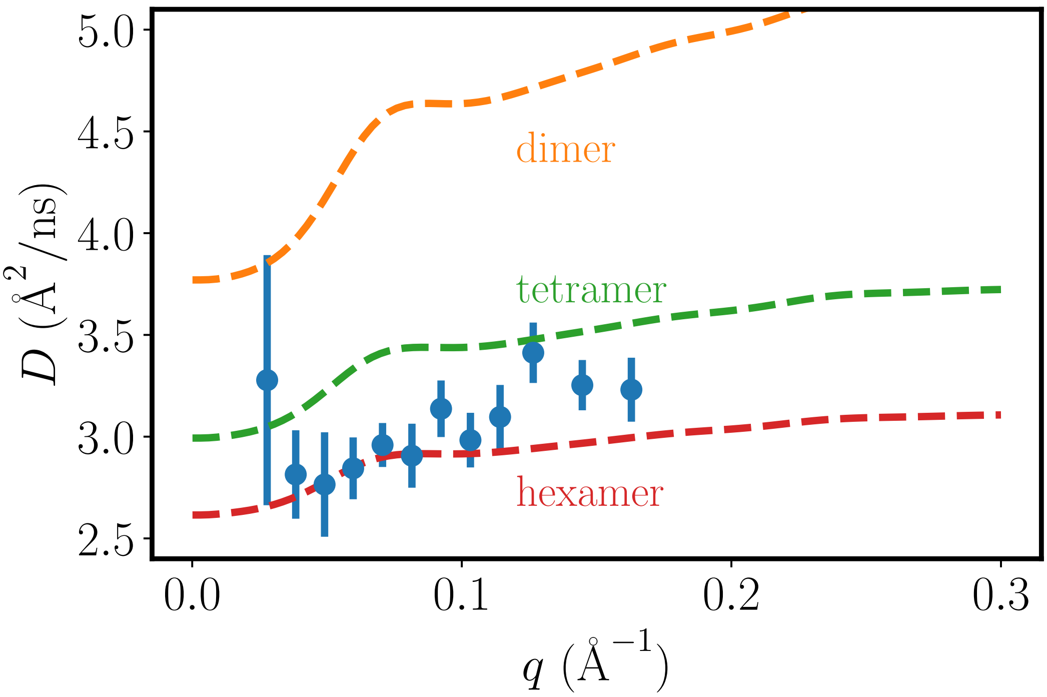

Figure S8: Comparing the rigid-body contribution from pure dimer, tetramer and hexamer solutions (dashed lines) with the experimental signature of $D_{\rm eff}$ (symbols) suggests the presence of oligomeric states. Given that the overall q signature seems conserved for the different oligomers, we do not use an assumed polydisperse system in our modeling, but restrict ourselves to a pure hexamer solution.

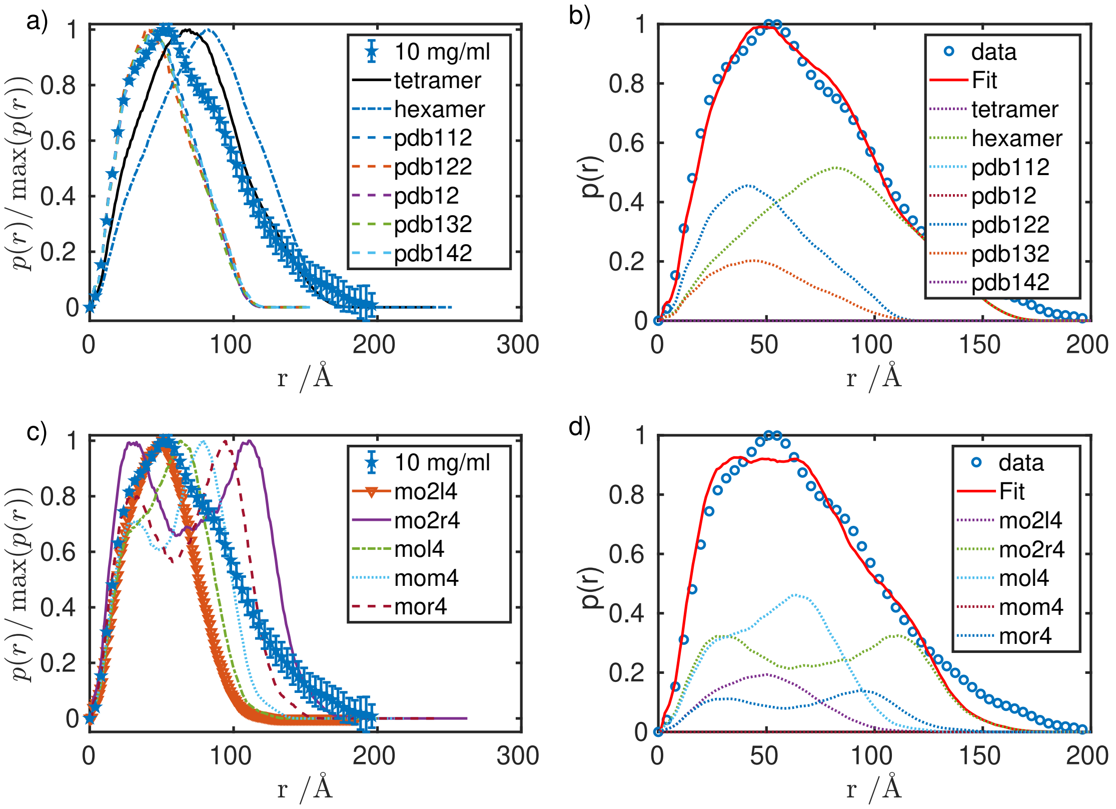

Figure S9: Pair distribution functions $p(r)$ of Hsp90: Stars and error bars (light blue) represent $p(r)$ determined from SANS data with SASView of a sample with 10 mg/ml. Additional lines correspond to calculated pair distribution functions based on structures from independent MD runs(plot a) and b)) or from structures opened to a different degree (plot c) and d)). For better visualization, the different distributions are either rescaled to their maximum value $\max(p(r))$ (plot a) and c)) or scaled in such a way, that their combination represents the experimentally determined distribution(plot b) and d).

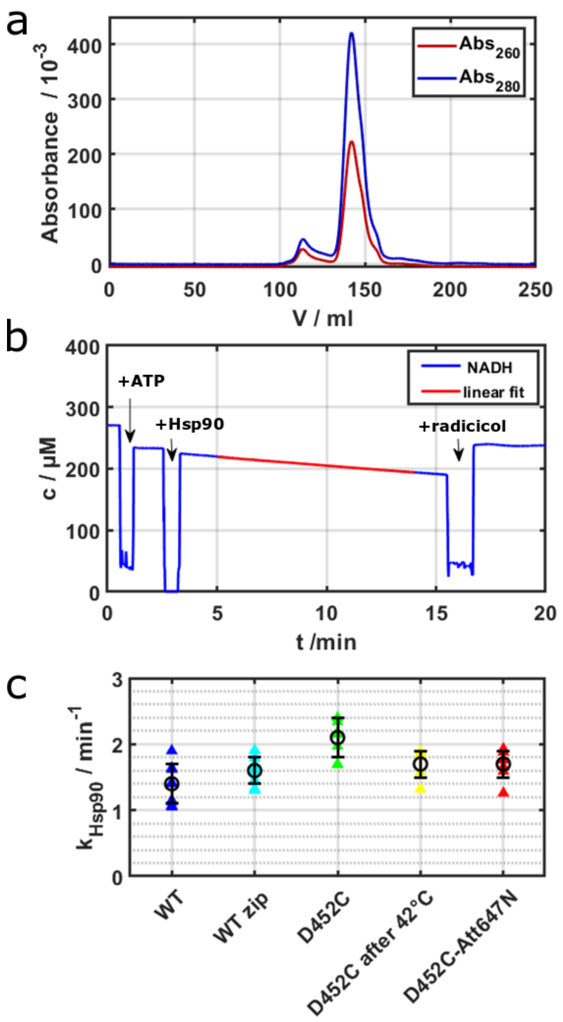

Figure S10: a) Representative SEC profile of the final purification step of yeast Hsp90 wildtype. The chromatogram shows two main fractions at 113 ml and 142 ml, respectively. The peak around 142 ml corresponds to the Hsp90 dimer and was pooled excluding the shoulder fraction. Size exclusion was performed on a cytiva Superdex 200 XK 26/600. b) ATPase assay of yeast Hsp90 wildtype. The assay couples ATP hydrolyzation to the oxidation of NADH to colorless NAD^+ which was monitored over time on a Lambda35 UV-Vis spectrometer. After addition of 2 mM ATP and 2 µM Hsp90 a linear decrease of the absorbance at 340 nm was followed. From the slope, an ATPase rate of $1.4 \pm 0.3\,\mathrm{min}^{-1}$ is obtained which is consistent with previous studies[1]. Final addition of the specific Hsp90 inhibitor radicicol stops Hsp90 ATP-turnover. Residual NADH consumption can be assigned to non-Hsp90 ATPAse and is excluded from the data. c) Summary of ATPase rates measured for yeast Hsp90 wildtype (WT), yeast Hsp90 with a C-terminally inserted coiled-coil motif, the cysteine variant D452C and the cysteine variant after the labeling procedure. As control the labeling procedure was performed with and without dye (D452C after 42°C and D452C-Atto647N, respectively). All ATPase assays resulted in similiar ATPase rates which makes Hsp90-related differences between fluorescence and neutron experiments as well as hindered bio-functionility unlikely.

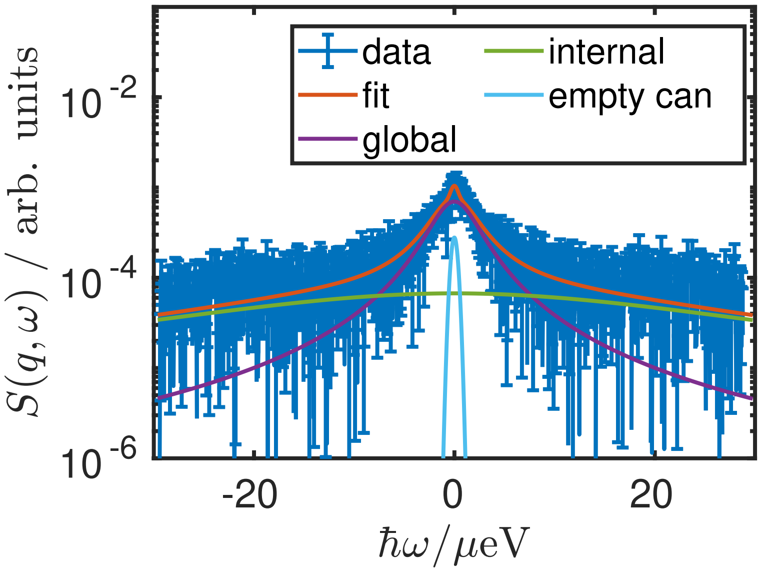

(a)

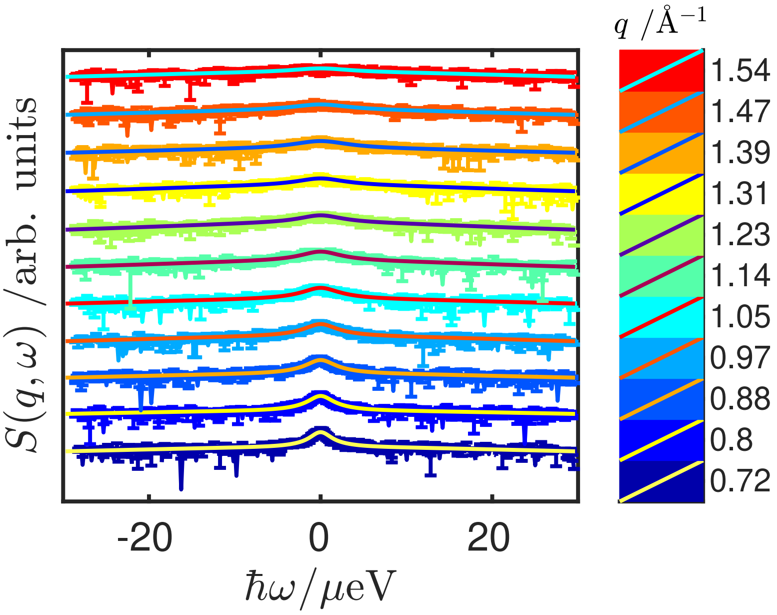

(b)

Figure S11: Fits of the neutron backscattering spectra of Hsp90 with 2 mM AMPPNP. a) fit with the different components as indicated in the legend for $q = 1$ Å^{-1}. b) Fit of the different q values. The data is shifted for better visibility and the q value of the spectra and the corresponding fit is color-coded on the right.

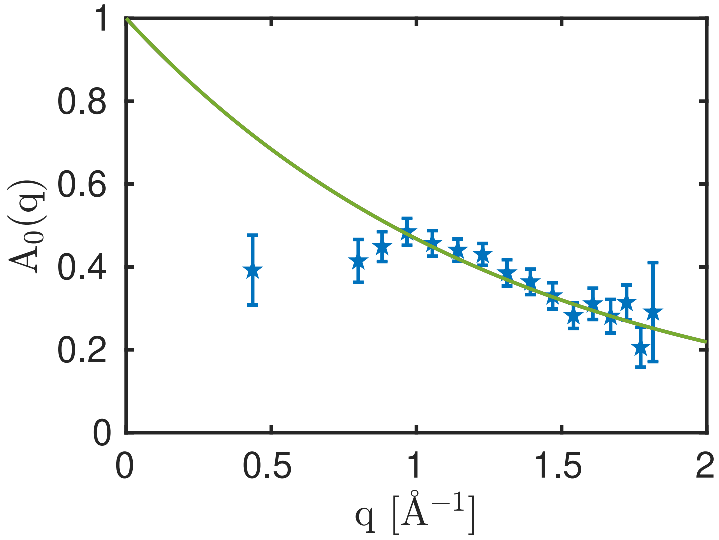

Figure S12: Elastic incoherent structure factor (EISF) obtained from the NBS analysis with corresponding fit (Eq. S2).

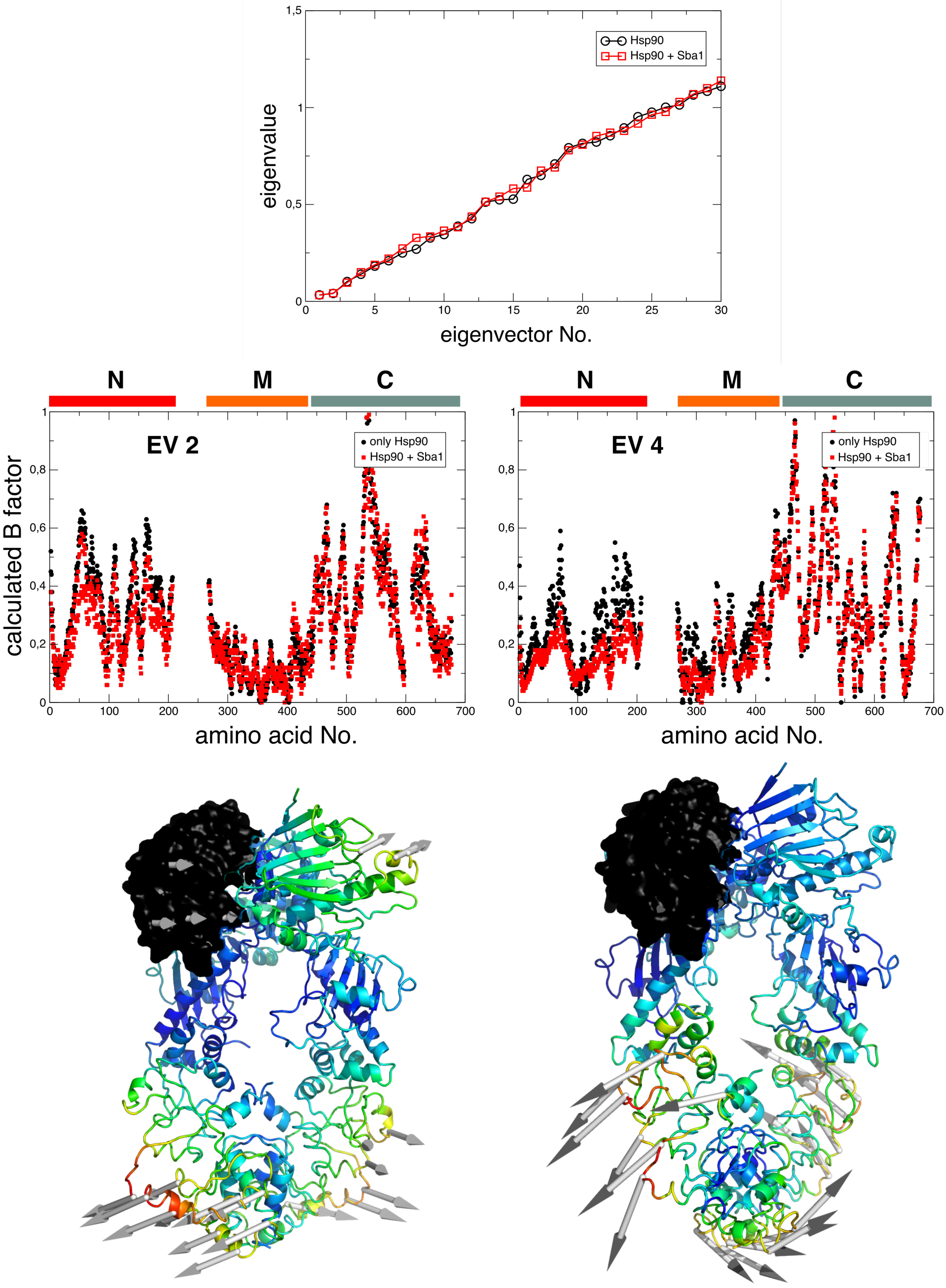

Figure S13: Anisotropic network model analysis. Top: eigenvalues of eigenvectors ordered according to lowest value / slowest oscillation (trivial first six modes not shown). Differences between Hsp90 with and without Sba1 are negligible. Middle: B factors per C_α atom calculated for exemplary normal modes 2 and 4. While mode 2 does not exhibit any significant differences between Hsp90 with and without Sba1, mode 4 exhibits clearly reduced dynamics around the N terminal domain. Bottom: Hsp90-Sba1 complex dynamics for normal modes 2 and 4, respectively. Sba1 as black surface. Hsp90 colored according to B factor with high factors in red, low factors in blue. Arrows indicate atoms with the highest contribution to / largest motion within the displayed normal modes.

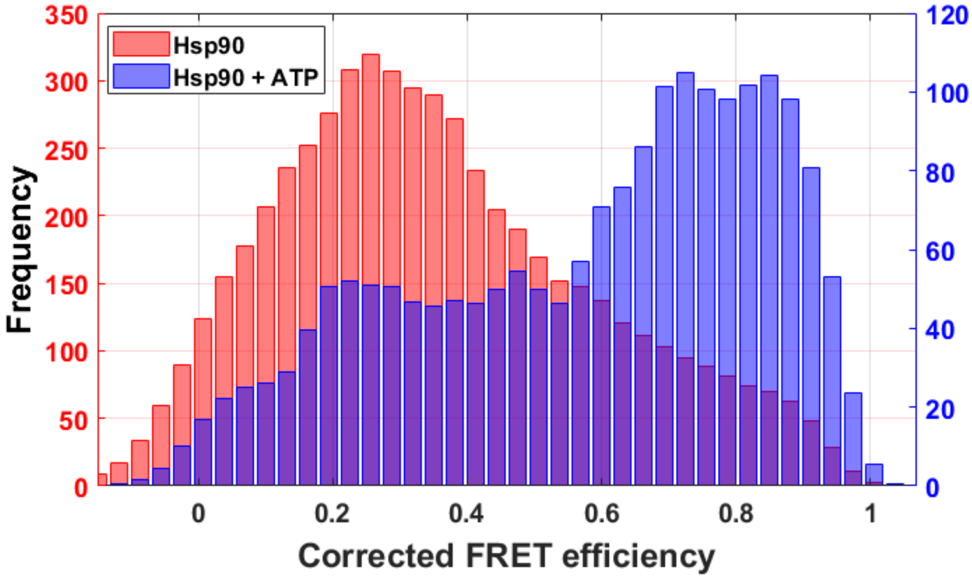

Figure S14: FRET efficiency histogram of Hsp90 in the apo condition (red) and with 2 mM ATP (blue) in D_2O based buffer. Hsp90 was labeled at position 452 with Atto550 and Atto647N as donor and acceptor, respectively. ATP depopulates the open conformational state of Hsp90 (here at E $\sim$ 0.28). This is the known behavior in H_2O-based buffer (see e.g. ref. [14] Fig. 3a) and gives evidence that D_2O does not have a crucial effect on the biologically relevant open-close dynamics of Hsp90.

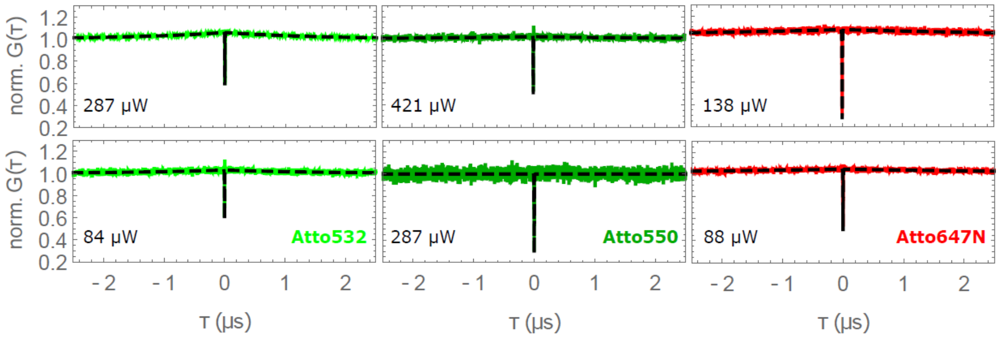

Figure S15: nsFCS data of Atto532 and Atto550 (correlation of DxD, respectivly) and Atto647N (correlation AxA) excited at different laser powers to reveal triplet kinetics. 'D' denotes donor signal after donor excitation, 'A' acceptor signal upon acceptor excitation. The analyses reveal a bunching mode on the 2-5 µs time scale which due to its laser-dependent weight can be ascribed to triplet kinetics. The indicated laser powers were measured by a power meter placed between the last dichroic mirror and the objective, measurements were performed with 150 µm pinholes. The fit results are given in Tab. S4.

2 Legends of Supplementary Movies

- **Supplementary Movie 1:** Morphing along the first eigenvector from the cartesian PCA. The displayed motion represents a morphing between the two structures appearing during simulations with minimal and maximal dot product of the cartesian coordinates of the protein and the first eigenvector. Hsp90 dimer displayed as cartoon representation.

- **Supplementary Movie 2:** Morphing along the second eigenvector from cartesian PCA. The displayed motion represents a morphing between the two structures appearing during simulations with minimal and maximal dot product of the cartesian coordinates of the protein and the second eigenvector. Hsp90 dimer displayed as cartoon representation.

- **Supplementary Movie 3:** Motion along the first eigenvector from cartesian PCA during one microsecond MD trajectory. The component of motion along the first eigenvector represents a fluctuating twist motion of the protein. Hsp90 dimer displayed as cartoon representation.

- **Supplementary Movie 4:** Motion along the second eigenvector from cartesian PCA during one microsecond MD trajectory. The component of motion along the second eigenvector represents a fluctuating twist motion of the protein. Hsp90 dimer displayed as cartoon representation.

3 Supplementary Methods

3.1 Sample Preparation

Gene expression and subsequent protein purification was performed as previously described [3]. pET derived expression plasmids contained the *hsp82* gene from baker's yeast with a cleavable N-terminal His_6-SUMO-tag. For fluorescent labeling a cysteine was introduced at position 61 and 452 via site-directed mutagenesis, respectively. *E. coli* BL21 Star (DE3) cells were transformed with the respective plasmid and cultivated in *lysogeny broth* medium supplemented with kanamycin at 37 °C. Expression was induced with 1 mM IPTG at OD_{600} between 0.6-0.8. Cells were harvested 4 h after induction and stored at -20 °C.

All buffers contained 40 mM HEPES and 150 mM NaCl at pH 7.5. Cleared lysate of His_6-SUMO-tagged yeast Hsp90 WT or D452C was applied to a HisTrap HP column (Cytiva, 10 ml) performing affinity chromatography (AC) and eluted with a gradient from 20 mM to 1 M imidazole in the buffer. For ion exchange chromatography (IEX), the salt concentration was adjusted to 30 mM NaCl for binding and 1 M for elution.

His_6-SUMO-tags were cleaved in presence of SenP protease while dialyzing against imidazole free buffer over night. A second HisTrap was applied to remove uncleaved fusion proteins and tags, leaving native like Hsp90 in the flow through. The flow through was diluted to 30 mM NaCl and applied to a HiTrap Q column (Cytiva, 10 ml). After elution with a NaCl gradient, target proteins were polished by a final size exclusion chromatography (SEC) step (S200, XK 26/600), concentrated and flash frozen in liquid nitrogen.

The final measurement buffer contained 40 mM HEPES, 150 mM KCl and 10 mM $MgCl_2$ dissolved in ultra pure H_2O with pH adjusted to 7.4 - 7.5 and is referred to as H1 buffer in the supplementary information. For neutron experiments D_2O instead of H_2O was used and HEPES was avoided to achieve a better sample signal.

3.2 Nanosecond Fluorescence Correlation Spectroscopy

Hsp90 nsFCS data sets were sufficiently described by Eq. 5 of the main manuscript. In Hsp90, a global bunching time on the ~150 ns component was obtained by global fitting optimising the fit to all three correlations: D×D, A×A and D×A. Here, 'A' represents the acceptor signal upon donor excitation. The autocorrelations D×D and A×A are always correlations between the perpendicular and parallel part of the signal which removes artefacts from detector afterpulsing.

The models used to fit the nsFCS data are described in [16] and result in Eq. 5 in the main manuscript.

3.3 Fluorescence lifetime and time-resolved single-molecule anisotropy analysis with PAM

Fluorescence lifetime analysis was performed using the software PAM [15]. For each single-molecule event FRET efficiency E, stoichiometry S and anisotropy r were de-

termined using Eq. 4-8 in main text, respectively. The Hsp90 data of consecutively measured 41h was analyzed sub-population specific for the closed A state. Obtained lifetimes for donor- and acceptor-only species of Hsp90 were compared to the fluorescence lifetime measured for free dye in ultra pure H_2O. Data with fits are shown in Fig. S4, fit results are given in Tab. S5. The determined lifetime of the dyes is in good agreement to the companies data sheet (Atto550: `https://www.atto-tec.com/fileadmin/user_upload/Katalog_Flyer_Support/ATTO_550.pdf`, Atto647N: `https://www.atto-tec.com/fileadmin/user_upload/Katalog_Flyer_Support/ATTO_647N.pdf`). The data in Fig. S4a and S4c (acceptor decay) can be well described with a single-exponential decay function with relaxation times of about 3 to 4 ns (namely in PAM 'Single Exponential'). For the data in Fig S4b and S4d (donor decay) a second relaxtion time is needed caused by light scattering or FRET process (Model Function in PAM named 'Biexponential'). The time-resolved anisotropy decays of the dyes and of Hsp90's closed A state were analyzed by the established fit models in PAM (Fig. S5 and Table S6) fitting globally the parallel and perpendicular decays of the detection channel. Here, the instrument response function IRF was considered in the analysis by performing a reconvolution. The decays of Atto647N and Atto550 were fitted accordingly to the lifetime analysis with 1 or 2 lifetimes and one rotational relaxation time of $\sim$0.3 ns for the dye self-rotation. For fitting the time-resolved single-molecule acceptor anisotropy decay of the closed A state of Hsp90 more rotational components are added successively. See Tab. S6 for all results.

3.4 Neutron Scattering

3.4.1 Neutron Backscattering

QENS data were reduced with Mantid [6] and further analyzed with Matlab 2020b (The MathWorks, Inc.) employing the Optimization and Curve Fitting Toolboxes.

Vanadium measurements were used to determine the resolution function and approximated by two Gaussian functions for each momentum transfer $\hbar q$.

To reduce possible cross-talking between different contributions in the fit, the solvent contribution (D_2O) to the scattering function was rescaled to account for the volume excluded by the proteins and subtracted from the scattering signal of the samples [7]. In addition, a global fit was performed fixing directly the q-dependence of the center-of-mass and internal diffusion linewidths (see below) parameters, thus reducing the number of optimization parameters.

This approach remedied the relatively low protein signal and while imposing more prior knowledge compared to other studies [7, 8]. The chosen approach nevertheless appears justified, since numerous previous studies have confirmed that the center-of-mass diffusion is of the Fickian type [9]. The scattering signal was described using two Lorentzian functions. The width of the first Lorentzian function represents the apparent short-time self diffusion and can be described by a Fickian diffusive process [9] $\gamma = D_{\mathrm{app}} q^2$ while the second Lorentzian function by averaging over all internal diffusive processes in the proteins is described by a q-independent width Γ. The contribution of the empty can was modeled with an elastic contribution. The incoherent scattering

function thus can be written as

$$S(q,\omega) = \mathscr{R}(q,\omega) \otimes [\beta_{EC}\delta(\omega) + \beta\left(A_0\mathscr{L}_\gamma(\omega) + (1-A_0)\mathscr{L}_{\gamma+\Gamma}(\omega)\right)] \quad \text{(S1)}$$

with A_0 being the q-dependent elastic incoherent structure factor (EISF) and β_{EC}, β being q-dependent scaling parameters. The EISF indirectly contains information on the local order within the protein via the geometry of the diffusive motion by which it is determined [9], [10]

Figure S11a shows the fit result of Equation S1 at $q = 1\,\text{Å}^{-1}$. The other q values are depicted in the SI in Figure S11b.

The short-time apparent diffusion coefficient D_{app}, being a combination of global short-time translational diffusion as well as global short-time rotational diffusion [9] was determined to a value of $D_{\text{app}} = (3.27 \pm 0.18)\ \frac{\text{Å}^2}{\text{ns}}$. The width Γ of the Lorentzian function describing the internal diffusion corresponds to a characteristic time $\tau = \frac{\hbar}{\Gamma} = (23.7 \pm 2.6)\,\text{ps}$. The EISF can be approximated by a diffusion around an equilibrium position of a potential with a Gaussian shape and an effective radius a [11]

$$A_0(q) = \exp\left(-\frac{(a \cdot q)^2}{5}\right) \quad \text{(S2)}$$

with $a = (1.95 \pm 0.07)$ Å. The corresponding fit is shown in Figure S12.

3.5 Pair Distance Distribution Functions

SANS measurements of a sample with $c_p = 10$ mg/ml in presence of AMPPNP were used to determine the pair distance distribution function $p(r)$. After a constant background subtraction, $p(r)$ was determined via the inversion algorithm of SASView [12]. The results with corresponding error bars are shown in Figure S9 (light blue stars). Additionally, pair distribution functions were calculated based on different Hsp90 structures using CaPP 3.12 [13]. $p(r)$ was calculated from 1 µs MD-trajectories of five independent runs, for pdb-based tetramers and hexamers (Fig. S8) and for different open Hsp90 structures (Fig. S8) obtained by smFRET-restrained modelling [14].

We find that the closed Hsp90 dimers alone are insufficient for a full description of our data. Especially at large distances the data deviates from $p(r)$ calculated based on the closed Hsp90 dimer structure. Therefore, the most likely explanation of our data is that some multimeric species is present in addition to Hsp90 dimers. In addition, likely a certain amount of open states is present, but this is below 10% in the presence of AMPPNP [5]. However, we are convinced that our first principle components show eigenmodes of the dimer, because the dimer is still the prevalent species and specific strong new modes from dimers of dimers or trimers of dimers are very unlikely.

4 Supplementary Tables

Summary of nsFCS fit results for FRET-labeled Hsp90

Table S1: Summary of substate-specific nsFCS fit results for all measured Hsp90 FRET pairs in presence of different additives and used burst search parameters. Samples were analyzed by selecting only bursts with population-specific FRET efficiencies (exact values are given in the respective figure captions). Label positions and used fluorophores are given in the order 'donor-acceptor'. 'Correlation' specifies the detection channels which were correlated to obtain the correlation function G(τ). DxD and AxA stand for the detection-specific cross-correlations of the parallel and perpendicular donor and acceptor channels, respectively. AxD stands for the colour-specific cross-correlation of the acceptor and donor detection channel. Fit parameters are a scaling factor a, the weight of the antibunching mode c_{ab}, the weight of the bunching modes c_b, antibunching time τ_{ab}, bunching time τ_b. τ_0 corrects for a small time delay between the detection channels, c is the used concentration. For the ΔT burst search method, ΔT was set to 150 µs and N_{max} was chosen by the 100 000 fold value of N_{min}. '# files' gives information about the amount of measured files which were analyzed for the given data set. Data was normalized for a.

sample	additives	data set ID	c / pM	correlation	a	c_{ab}	c_b	τ_{ab} / ns	τ_b / ns	τ_0 / ns	# files (1 h each)	N_{min}
452-452 (closed A)	AMPPNP	1A	500	A x A	0.993239	0.876413	0.0918451	1.94158	143.469	-0.55815	23	75
				D x D	1.0117	0.624809	0.142681	1.59451		-0.786949		
				A x D	0.999438	0.156373	0.10214	-		-1.9537		
		1B	1000	A x A	1.00612	0.804534	0.0868459	2.30609	141.26	-0.57446	60	75
				D x D	1.00405	0.601682	0.135063	1.54818		-0.646977		
				A x D	1.0086	0.157981	0.0988546	-		-2.30947		
		1C	500	A x A	1.00639	0.853536	0.0964612	2.32502	151.447	-0.620113	68	75
				D x D	1.02963	0.651451	0.16946	1.74905		-0.660123		
				A x D	1.00242	0.187743	0.116784	-		-2.54387		
		1D	200	A x A	1.00015	0.144593	0.104159	2.1334	144.593	-0.416017	7	50
				D x D	1.01184	0.854236	0.176857	1.23432		-0.532229		
				A x D	1.01854	0.220895	0.122864	-		-3.0292		
		1E	500	A x A	1.00813	0.762268	0.0851402	2.34297	157.679	-0.529907	20	75
				D x D	1.0178	0.557979	0.128854	1.8827		-1.24674		
				A x D	1.01367	0.123551	0.0862635	-		-2.43242		
		1F	500	A x A	0.98119	0.870269	0.0915966	2.22761	157.495	-0.618933	41	75
				D x D	0.985911	0.596948	0.147285	1.47875		-0.833088		
				A x D	1.00906	0.162648	0.10513	-		-2.21447		

sample	additives	data set ID	c / pM	correlation	a	c_{ab}	c_b	τ_{ab} / ns	τ_b / ns	τ_0 / ns	# files (1 h each)	N_{min}
452-452 (closed A)	AMPPNP, Sba1	2A	500	A x A	1.01443	0.796166	0.0906997	2.25185	161.357	-0.60889	45	75
				D x D	1.01055	0.550961	0.137895	1.76084		-0.861216		
				A x D	1.0058	0.150636	0.105642	-		-1.7406		
		2B	500	A x A	1.00135	0.792354	0.0855014	2.05173	141.549	-0.587925	51	75
				D x D	1.02783	0.582186	0.122324	1.45666		-0.607672		
				A x D	0.994655	0.197553	0.102194	-		-2.46525		
452-452 (open)	None	3A	200	A x A	1.04206	0.800256	0.204349	2.19163	195.036	-0.402164	9	50
				D x D	1.01106	0.732706	0.161751	2.95456		-0.682633		
				A x D	0.980497	0.427893	0.176938	-		-1.20535		
		3B	500	A x A	1.08518	0.874015	0.178639	1.78882	175.697	-0.701289	20	75
				D x D	1.0067	0.72561	0.153483	3.27738		-0.789758		
				A x D	1.00323	0.342324	0.160412	-		-0.628995		
		3C	200	A x A	1.03744	0.976421	0.180863	1.41979	191.08	-0.353584	11	50
				D x D	1.01868	0.774195	0.16164	3.07301		-0.61512		
				A x D	1.0542	0.44084	0.178463	-		-0.37884		
61-61 (closed)	AMPPNP	4A	200	A x A	1.01693	0.768963	0.168031	1.90024	167.912	-0.482739	62	75
				D x D	1.00377	0.687199	0.144084	3.34054		-0.763617		
				A x D	1.01146	0.41601	0.228008	-		-0.661715		
		4B	200	A x A	1.0205	0.860816	0.177141	1.92113	162.962	-0.502622	26	75
				D x D	1.01519	0.699792	0.149212	3.50155		-0.824155		
				A x D	0.998042	0.430123	0.240705	-		-0.706309		
61-61 (closed)	AMPPNP, Sba1	5A	200	A x A	1.01487	0.683559	0.170908	2.20814	189.354	-0.812286	18	75
				D x D	0.99783	0.739065	0.149582	3.10003		-0.741608		
				A x D	1.00602	0.438791	0.249593	-		-1.1814		
		5B	200	A x A	1.0047	0.775183	0.172339	1.93139	186.653	-0.609397	65	75
				D x D	1.00674	0.775183	0.153021	3.47673		-0.848712		
				A x D	0.998564	0.429338	0.242301	-		-0.772804		

Fit results of single-molecule time-resolved anisotropy analysis

Table S2: Acceptor anisotropy analysis at Hsp90 position 452. The Hsp90 FRET pair 452-452 was labeled with Atto550 and Atto647N and measured with AMPPNP at 22°C. FRET vs. stoichiometry analysis was used to assign conformational sub-states of Hsp90 (open, closed A, closed B). Double labelled Hsp90's are necessary to assign the conformational sub-states. Data were analyzed using the *cone-in-cone* model [2]: ρ_{dye} describes free dye rotation, ρ_{local} the rotation of structural elements to which Atto647N was attached to (e.g. a loop) and ρ_{global} the global rotation of the overall protein.

state	channel	r_0	A_{dye}	A_{local}	ρ_{dye} / ns	ρ_{local} / ns	ρ_{global} / ns
open	AA	0.4	0.41 ± 0.03	0.46 ± 0.06	0.35 ± 0.05	3.2 ± 0.8	52 ± 32
closed A	AA	0.4	0.40 ± 0.03	0.22 ± 0.03	0.35 ± 0.05	2.8 ± 0.3	52 ± 32
closed B	AA	0.4	0.31 ± 0.04	0.52 ± 0.05	0.35 ± 0.05	2.5 ± 1.1	52 ± 32

Fit results of MD-based accessible dye volume auto-correlations

Table S3: Fit results of MD-based accessible dye volume correlations analysis. Accessible dye volumes were auto-correlated for different Hsp90 positions based on 1 µs-MD traces with AMPPNP (A = chain A, B = chain B).

correlation	A_1	A_2	τ_1 / ns	τ_2 / ns	R^2
A61 x A61	$(69 \pm 6) \cdot 10^9$	$(66.8 \pm 0.4) \cdot 10^9$	$3 \cdot 10^{-5} \pm$NaN	103 ± 1	0.97
B61 x B61	$(120 \pm 7) \cdot 10^9$	$(70.0 \pm 0.6) \cdot 10^9$	$0.16 \pm$NaN	72 ± 1	0.95
A452 x A452	$(120 \pm 10) \cdot 10^9$	$(49.5 \pm 0.7) \cdot 10^9$	$3 \cdot 10^{-5} \pm$NaN	100 ± 2	0.78
B452 x B452	$(120 \pm 5) \cdot 10^9$	$(70.0 \pm 0.8) \cdot 10^9$	$0.9 \pm$NaN	75 ± 1	0.92

Summary of nsFCS fit results for free dyes at different excitation powers

Table S4: nsFCS results of Atto532, Atto550 and Atto647N in H1 buffer at different excitation powers. Laser powers were determined by a power meter placed between the objective and the last dichroic mirror. 'Correlation' specifies the detection channels which were correlated to obtain the correlation function G(τ). D×D and A×A stand for the cross-correlations of the parallel and perpendicular donor and acceptor channels, respectively. Cross-correlation of different polarisation channels removed artefacts resulting from detector afterpulsing. Eq. 5 of the main manuscript was used as fit model which includes a scaling factor (a), the weight of the antibunching mode (c_{ab}), the weights of the bunching mode (c_b), the antibunching time (τ_{ab}) and bunching time (τ_b), respectively. τ_0 corrects for a small time delay between the detection channels. The data was analyzed from 1 ns to 2.5 µs. +/- values represent the standard fit error.

fluorophore	excitation power / µW	correlation	a	c_{ab}	c_b	τ_{ab} / ns	τ_b / µs	τ_0 / ns
Atto532	84	D×D	76.0±0.2	0.58±0.017	0.031±0.002	1.68±0.07	1.6±0.2	-0.64±0.03
	287	D×D	12.20±0.010	0.598±0.005	0.0608±0.0008	2.00±0.02	1.60±0.05	-0.57±0.012
Atto550	287	D×D	242.4±0.11	0.94±0.03	-	2.1±0.10	-	-0.59±0.05
	421	D×D	22.35±0.06	0.69±0.02	0.020±0.003	1.72±0.06	1.8±0.5	-0.63±0.03
Atto647N	88	A×A	15.96±0.02	0.59±0.010	0.047±0.0015	2.67±0.06	5	0.11±0.03
	138	A×A	46±1	0.83±0.011	0.08±0.03	3.21±0.06	5±2	0.09±0.03

Lifetime analysis fit results for free dye, Hsp90 donor-only and acceptor-only populations.

Table S5: Single-molecule data were described either with a single-exponential or a bi-exponetial reconvolution fit. For data analysis, PAM was used. IRF and background were carefully aligned to the TCSPC histograms. In case of analyzed TCSPC histograms of the DD channel, a second lifetime was needed to describe the data caused by scattered light or the FRET process. τ_1 and τ_2 represent the fluorescence lifetime in ns.

sample	channel	τ_1 / ns	τ_2 / ns	fraction a_1	I_0 / counts	$\mathcal{X}^2_{\text{red}}$
Atto647N	AA	3.13 ± 0.02	-		23718.42	1.23
Atto550	DD	3.28 ± 0.08	1.14 ± 0.05	0.65 ± 0.02	87945.64	1.33
452-452 closed A	AA	3.838 ± 0.008	-		55137.30	1.22
452-452 closed A	DD	1.95 ± 0.02	0.48 ± 0.02	0.597 ± 0.009	80606.10	2.76

Fit results of single-molecule time-resolved anisotropy analysis by PAM

Table S6: TRA of donor and acceptor decay of Atto550 and Atto647N and TRA analysis of acceptor decay of labelled Hsp90 at positions 452. The Hsp90 FRET pair 452-452 was labeled with Atto550 and Atto647N and measured with AMPPNP at 22°C. FRET vs. stoichiometry analysis was used to assign conformational sub-states of Hsp90 (open, closed A, closed B). Data were analyzed using the established models in PAM with different amount of rotation components: ρ_i describes the different rotational decay times. Fit models are named in PAM as following: 1a = 'Fit Anisotropy', 1b ='Fit Anisotropy (2 exp lifetime)', 2 ='Fit Anisotropy (2 exp rot)', 3 ='Fit Anisotropy (4 exp lifetime, 4 exp rot)'. Note that for fit model 3 the parameter range were specified for 3 rotational components and one lifetime decay and the other parameters were set to zero, fraction 1 a_1 was set to 1.

sample	chan.	Fit model	τ_1 / ns	τ_2 / ns	a_1	ρ_1 /ns	ρ_2 /ns	ρ_3 / ns	r_0	r_2	r_3	r_∞	$\mathcal{X}^2_{\text{red}}$
Atto647N	AA	1a	3.19 ± 0.02	-	-	0.27± 0.01	-	-	0.36 ± 0.02	-	-	0.002 ± 0.002	1.16
Atto550	DD	1b	3.39 ± 0.06	1.22 ± 0.04	0.61 ± 0.02	0.301 ± 0.009	-	-	0.251 ± 0.007	-	-	0.0000 ± 0.0010	1.18
Hsp90 closed A	AA	1a	3.811 ± 0.010	-	-	1.05 ± 0.05	-	-	0.178 ± 0.003	-	-	0.052 ± 0.002	1.48
Hsp90 closed A	AA	2	3.839 ± 0.010	-	-	0.54 ± 0.05	6.6 ± 0.7	-	0.207 ± 0.007	0.090 ± 0.004	-	-	1.21
Hsp90 closed A	AA	3	3.81 ± 0.04	-	1	0.14 ± 0.05	1.3 ± 0.4	44 ± 67	0.30 ± 0.04	0.086 ± 0.010	0.06 ± 0.01	-	2.94

5 Supplementary Note

Correlations of changes in accessible volumes of dyes

Protein dynamics can cause changes in the accessible volume (AV) of the dyes and therefore influence the dyes' photophysics. We determined the AV for every time step in the MD simulations and correlated the AVs, which resulted in correlation times of ~ 100 ns. Changes in AVs can affect the mean positions of the dyes and therefore the rate of the energy transfer (see Fig. S16a). Changes in the rate of energy transfer would affect the photon counts from the dyes and therefore dynamic changes of the AVs would be also directly reflected in both, auto- and cross-correlations from nsFCS. Specifically in the cross-correlation this would lead to an anti-correlated signal, which is not the prevalent signal in our data. Another mechanism is static or dynamic quenching of the dyes, which is likely the dominant signal in our data, because we see a reduction of the lifetime of the donor when attached to Hsp90 (Tab. S5) and in the nsFCS data (Tab. S1). This is further supported by known quencher (for example tryptophan (W) or tyrosine (Y)) in close proximity to the AVs of the used dyes in the closed conformation of yeast Hsp90 (PDB: 2cg9, AVs calculated by FPS, see Fig. S16b-d. In addition, the photophysics of the dyes might be slaved to the dynamics of the protein surface by temporary attachment or other restrictions of their motion, as labels are large heterocycles with a considerable hydrophobic surface.

The observed dynamic changes in photophysics do not only reflect local dynamics of the dyes for the following reasons: Neutron scattering is consistent with molecule spanning dynamics on this time scale and we observe similar dynamics for different labeling positions. Especially the latter is a strong indication for our interpretation that changes in AV reflect molecule spanning dynamics on the 100 ns time scale. Because it is very unlikely that local static quenching caused by dye movement is at a similar time scale for all different labeling positions and for the different dyes. We do not exclude additional contributions to the nsFCS signals. Nevertheless, because of the good agreement to the simulations and neutron scattering experiments we are convinced that in our case changes in the AV dominate the nsFCS signals.

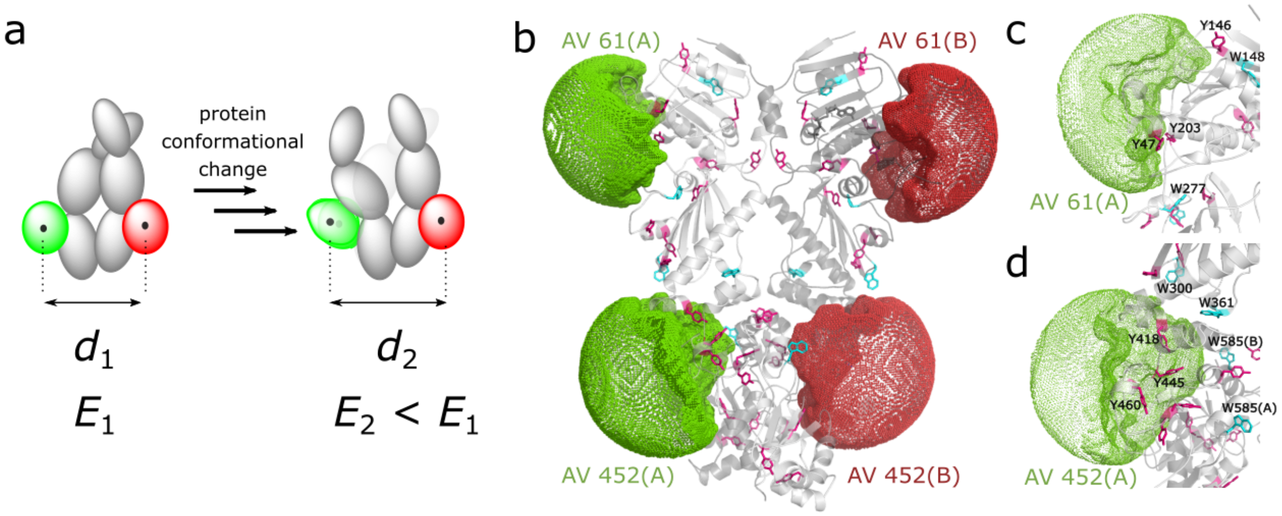

Figure S16: a) Schematic of accessible volumes (AVs) of two FRET dyes before and after a hypothetical conformational change. The distances between the centers of the AVs (d_1, d_2) and therefore the FRET efficiencies (E_1, E_2) might change during the conformational change. b) Illustration of Hsp90 in the closed conformation (PDB 2cg9) including AVs for the labeling positions 61 and 452. Possible fluorescence quenchers such as tryptophan (five per monomer) and tyrosine (20 per monomer) are highlighted in cyan and magenta, respectively. c-d) Close-ups of the AVs of both labeling positions depict the tyrosine and tryptophan residues that are in close proximity to the respective AVs of the dyes therefore representing the most likely candidates for quenching.

References

[1] H. Girstmair, F. Tippel, A. Lopez, K. Tych, F. Stein, P. Haberkant, P. W. N. Schmid, D. Helm, M. Rief, M. Sattler, and J. Buchner. The hsp90 isoforms from s. cerevisiae differ in structure, function and client range. *Nature communications*, 10 (1):3626, 2019.

[2] G. F. Schröder, U. Alexiev, and H. Grubmüller. Simulation of fluorescence anisotropy experiments: probing protein dynamics. *Biophysical journal*, 89(6): 3757–3770, 2005.

[3] S. Schmid and T. Hugel. Controlling protein function by fine-tuning conformational flexibility. *eLife*, 9:1013, 2020.

[4] D. A. Rutz, Q. Luo, L. Freiburger, T. Madl, V. R. I. Kaila, M. Sattler, and J. Buchner. A switch point in the molecular chaperone hsp90 responding to client interaction: Supplementory information. *Nature communications*, 9(1):1472, 2018.

[5] S. Wolf, B. Sohmen, B. Hellenkamp, J. Thurn, G. Stock, and T. Hugel. Hierarchical dynamics in allostery following ATP hydrolysis monitored by single molecule FRET measurements and MD simulations. *Chem. Sci.*, 12:3350–3359, 2021.

[6] O. Arnold, J. Bilheux, J. Borreguero, A. Buts, S. Campbell, L. Chapon, M. Doucet, N. Draper, R. F. Leal, M. Gigg, V. Lynch, A. Markvardsen, D. Mikkelson, R. Mikkelson, R. Miller, K. Palmen, P. Parker, G. Passos, T. Perring, P. Peterson, S. Ren, M. Reuter, A. Savici, J. Taylor, R. Taylor, R. Tolchenov, W. Zhou, and J. Zikovsky. Mantid—data analysis and visualization package for neutron scattering andμSR experiments. *Nuclear Instruments and Methods in Physics Research Section A: Accelerators, Spectrometers, Detectors and Associated Equipment*, 764: 156–166, November 2014.

[7] C. Beck, M. Grimaldo, F. Roosen-Runge, M. Braun, F. Zhang, F. Schreiber, and T. Seydel. Nanosecond Tracer Diffusion as a Probe of the Solution Structure and Molecular Mobility of Protein Assemblies: The Case of Ovalbumin. *J. Phys. Chem. B*, 122(35):8343–8350, 2018.

[8] M. Grimaldo, H. Lopez, C. Beck, F. Roosen-Runge, M. Moulin, J. M. Devos, V. Laux, M. Härtlein, S. Da Vela, R. Schweins, A. Mariani, F. Zhang, J.-L. Barrat, M. Oettel, V. T. Forsyth, T. Seydel, and F. Schreiber. Protein Short-Time Diffusion in a Naturally Crowded Environment. *J. Phys. Chem. Lett.*, 10(8):1709–1715, 2019.

[9] M. Grimaldo, F. Roosen-Runge, F. Zhang, F. Schreiber, and T. Seydel. Dynamics of proteins in solution. *Quarterly Reviews of Biophysics*, 52:e7, 1, 2019.

[10] F. Volino and A. Dianoux. Neutron incoherent scattering law for diffusion in a potential of spherical symmetry: general formalism and application to diffusion inside a sphere. *Molecular Physics*, 41(2):271–279, 1980.

[11] F. Volino, J.-C. Perrin, and S. Lyonnard. Gaussian model for localized translational motion: application to incoherent neutron scattering. *The Journal of Physical Chemistry B*, 110(23):11217–11223, 2006.

[12] SasView, `http://www.sasview.org`.

[13] CaPP, `github.com/Niels-Bohr-Institute-XNS-StructBiophys/CaPP`.

[14] B. Hellenkamp, P. Wortmann, F. Kandzia, M. Zacharias, and T. Hugel. Multidomain structure and correlated dynamics determined by self-consistent fret networks. *Nature methods*, 14(2):174–180, 2017.

[15] W. Schrimpf., A. Barth, J. Hendrix, and D. C. Lamb. PAM: A Framework for Integrated Analysis of Imaging, Single-Molecule, and Ensemble Fluorescence Data. *Biophysical journal*, 71518–1528, 2018.

[16] M. Nüesch, M. Ivanovic, J.-B. Claude, D. Nettels, R.B. Best, J. Wenger and B. Schuler. Single-molecule Detection of Ultrafast Biomolecular Dynamics with Nanophotonics. *Journal of the American Chemical Society*, 144(1) 52-56, 2022.
